# Crystal structure of the catalytic domain of HIV-1 restriction factor APOBEC3G in complex with ssDNA

**DOI:** 10.1038/s41467-018-04872-8

**Published:** 2018-06-25

**Authors:** Atanu Maiti, Wazo Myint, Tapan Kanai, Krista Delviks-Frankenberry, Christina Sierra Rodriguez, Vinay K. Pathak, Celia A. Schiffer, Hiroshi Matsuo

**Affiliations:** 1 Basic Science Program, Leidos Biomedical Research, Inc., Frederick National Laboratory for Cancer Research, Frederick, MD 21702 USA; 2 Viral Mutation Section, HIV Dynamics and Replication Program, Center for Cancer Research, National Cancer Institute at Frederick, Frederick, MD 21702 USA; 3 Department of Biochemistry and Molecular Pharmacology, University of Massachusetts Medical School, Worcester, MA 01655 USA

## Abstract

The human APOBEC3G protein is a cytidine deaminase that generates cytidine to deoxy-uridine mutations in single-stranded DNA (ssDNA), and capable of restricting replication of HIV-1 by generating mutations in viral genome. The mechanism by which APOBEC3G specifically deaminates 5′-CC motifs has remained elusive since structural studies have been hampered due to apparently weak ssDNA binding of the catalytic domain of APOBEC3G. We overcame the problem by generating a highly active variant with higher ssDNA affinity. Here, we present the crystal structure of this variant complexed with a ssDNA substrate at 1.86 Å resolution. This structure reveals atomic-level interactions by which APOBEC3G recognizes a functionally-relevant 5′-TCCCA sequence. This complex also reveals a key role of W211 in substrate recognition, implicating a similar recognition in activation-induced cytidine deaminase (AID) with a conserved tryptophan.

## Abstract (web summary)

APOBEC3G (A3G) is a single-stranded DNA (ssDNA) cytidine deaminase that restricts HIV-1. Here the authors provide molecular insights into A3G substrate recognition by determining the 1.86 Å resolution crystal structure of its catalytic domain bound to ssDNA.

## Introduction

Human APOBEC (“apolipoprotein B mRNA editing enzyme, catalytic polypeptide”) proteins are single-stranded DNA (ssDNA) cytidine deaminases that catalyze the Zn-dependent deamination of a deoxy-cytidine, generating deoxy-uridine^1^. The APOBEC family includes APOBEC1, APOBEC2, APOBEC3, APOBEC4, and activation-induced cytidine deaminase (AID)^1^. Among them, APOBEC3 proteins are genetically expanded in humans in response to the evolution of pathogens^2,3^. As a result of this expansion, humans contain seven APOBEC3 proteins (APOBEC3A, 3B, 3C, 3D, 3F, 3G, and 3H), which are all encoded on chromosome 22^4^. APOBEC3G (A3G) restricts human immunodeficiency virus type 1 (HIV-1)^5–9^, a finding that prompted extensive studies of APOBEC3 protein restriction of retroviruses and retrotransposons^10–13^. Of the seven human APOBEC3 proteins, A3D, A3F, A3G, and A3H can restrict HIV-1, and hypermutation of the virus genomes by their deamination activity is the primary mechanism by which these A3 proteins restrict HIV-1^10–13^. The APOBEC3 proteins catalyze the deamination of deoxy-cytidine introducing C-to-U modifications in newly synthesized (−)DNA strands of the virus genome, which results in G-to-A mutations in (+)DNA as U is used as a template during (+)DNA strand synthesis^14^. Although, there are many reports of deaminase-independent HIV restriction by APOBEC3 proteins, the significance of deaminase-independent mechanisms remains elusive and requires further study^15^.

HIV-1 has developed a mechanism against APOBEC3 proteins by using one of its accessory proteins, namely viral infectivity factor or Vif^16^. Vif physically interacts with HIV-relevant APOBEC3 proteins, and assembles host cellular proteins including an E3 ubiquitin ligase to trigger degradation of the APOBEC3 proteins through the ubiquitin–proteasome pathway^16^. For A3D, A3F, and A3G, which contain two Zn^2+^-binding motifs/domains, the catalytically inactive N-terminal domain (NTD) binds Vif as well as RNA, DNA, and other viral proteins^17^. The C-terminal domain (CTD) of these APOBEC3 proteins is catalytically active, containing Zn^2+^-binding motif HxE-x_23–28_-C-x_2–4_-C. The catalytic mechanism of cytosine/cytidine deamination has been studied biochemically and structurally using deaminases from *Escherichia coli* and yeast. Briefly, the hydroxide ion generated from a water molecule chelating Zn^2+^ attacks the C4 atom of cytosine, then the hydrogen is transferred to the carboxylate group of glutamic acid from the Zn^2+^-binding motif; this hydrogen is ultimately transferred to the product ammonia^18–20^.

Although all APOBEC3 proteins deaminate cytidines in ssDNA, they show differences in preferred hotspot sequences as 5ʹ-CC for A3G and 5ʹ-TC for other A3s (A3A can deaminate 5ʹ-CC albeit to a lesser extent)^21^. A3G’s deamination mechanism may be more complicated than that of other A3 proteins because several groups have reported that A3G deaminates 5ʹ-CC hotspots processively from the 3ʹ-end to the 5ʹ-end of ssDNA^22^.

Three-dimensional structures of APOBEC proteins have emerged in the last 10 years as our laboratories^23–29^ along with others^22,30–40^ have solved nuclear magnetic resonance (NMR) and crystal structures of single domains of human APOBEC proteins. These structures are similar as they share the same secondary structure, including six helices and five β-strands, and one H_A_E_x28_C_x2–4_C zinc-binding motif. We and others have proposed several alternative ssDNA binding surfaces for A3G-CTD based on NMR and crystal structures of apo-form CTDs^23,30,31,35^, yet none of these models are convincing because they lack atomic-level information of interactions between ssDNA and protein. Most recently, the crystal structures of A3A in complex with ssDNA containing a 5ʹ-TC deamination motif have been reported by us and others^29,39^. These A3A–ssDNA co-crystal structures revealed the interactions between A3A and the 5ʹ-TC motif, and structural similarity with the crystal structure of *Staphylococcus aureus* tRNA adenosine deaminase (TadA) in complex with RNA^29,39^.

In this study, we present the co-crystal structure of the A3G-CTD and ssDNA at 1.86 Å resolution. To overcome A3G-CTD weak DNA-binding affinity, we generated a catalytically enhanced variant of A3G-CTD that binds ssDNA stronger than wild-type. This A3G-CTD variant was co-crystallized with a 9 nucleotides ssDNA containing a 5ʹ-TCCCA target sequence with all nine nucleotides well resolved in the structure. The nucleotides within the 5ʹ-TCCCA target sequence show numerous interactions with protein, explaining the nucleotide specificity preferences. Furthermore, the backbone architecture of the protein changed upon ssDNA binding, enabling the target sequence to fit. These results provide fundamental insights into the mechanisms by which APOBEC3s recognize their specific substrate sequences.

## Results

### Generation of a Hyperactive A3G-CTD Variant

**Fig. 1:**
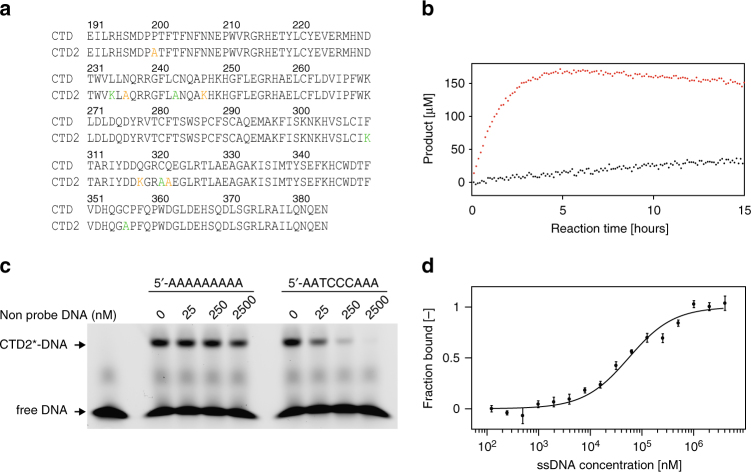
Generation of a hyperactive A3G-CTD variant. **a** Amino acid residues of wild-type A3G-CTD and the catalytically hyper-active variant, namely CTD2, are aligned. 2K3A substitutions (L234K, C243A, F310K, C321A, and C356A) are colored green, and the additional five substitutions (P200A, N236A, P247K, Q318K, and Q322A) are colored orange. **b** Real-time NMR deamination assay. The product (5ʹ-AATCCdeoxy-UAAA) concentration as a function of reaction time is plotted for CTD2 (red dots) and wild-type A3G-CTD (black dots) deamination at pH 7.5 with 200 nM protein and 200 µM 5ʹ-AATCCCAAA substrate. For CTD2, the first reaction reached completion within 5 h, and the second deamination (5ʹ-AATCCdeoxy-UAAA to 5ʹ-AATCdeoxy-Udeoxy-UAAA) started, thus decreasing the concentration of the initial product. **c** EMSA for binding of CTD2* to the 9nt ssDNA (5′-AATCCCAAA-6-FAM). CTD2*-DNA indicates position of the CTD2*–ssDNA complex, and free DNA indicates the position of protein-free ssDNA. Fluorescent-unprobed 9nt polyA (5′-AAAAAAAAA) or fluorescent-unprobed 9nt ssDNA (5′-AATCCCAAA) were added with incremental amounts in lanes 3 (25 nM), 4 (250 nM), and 5 (2500 nM) or 7 (25 nM), 8 (250 nM), and 9 (2500 nM), respectively. Uncropped gel is shown in Supplementary Fig. 5. **d** Each dot represents microscale thermophoresis (MST) measurement of a mixture containing fluorescent-labeled CTD2* (50 nM) and the 9nt ssDNA 5ʹ-AATCCCAAA at various concentrations including 0.12 µM, 0.24 µM, 0.48 µM, 0.97 µM, 1.95 µM, 3.90 µM, 7.81 µM, 15.62 µM, 31.25 µM, 62.5 µM, 125 µM, 250 µM, 500 µM, 1 mM, 2 mM, and 4 mM. Three independent MST experiments were performed, and bars of data points represent standard error of *n* = 3 measurements

Although the CTD of A3G is catalytically active in vitro^31,41^, detecting strong ssDNA-binding or purifying stable A3G-CTD–ssDNA complex has been challenging. To overcome this challenge, we designed and generated variants that are catalytically more active than the wild-type protein by introducing amino acid substitutions. The rationalization was that catalytically hyperactive variants may have increased affinity for the substrate, while retaining the intact structure and catalytic mechanism. A similar strategy worked well for us to generate a soluble A3G-CTD variant, namely CTD-2K3A, enabling our determination of the solution NMR structure^23^. CTD-2K3A contains five amino acid substitutions including L234K, C243A, F310K, C321A, and C356A, and these substitutions alter neither catalytic activity, structure, nor HIV-1 restriction function, but increased solubility^23,25^. Starting from CTD-2K3A, five additional substitutions were made, including P200A, N236A, P247K, Q318K, and Q322A. To increase the basicity of the region near the active site, we introduced lysine residues in loop3 and loop7; P247K and Q318K were chosen as they enhanced catalytic activity. Those lysine substitutions were combined with the other three substitutions, which we previously showed to increase catalytic activity^23^. This variant, hereafter called CTD2, spans residues 191–384 of A3G, and contains P200A, L234K, N236A, C243A, P247K, F310K, Q318K, C321A, Q322A, and C356A substitutions (Fig. 1a).

The initial reaction speed of deamination of CTD2 was compared with that of wild-type A3G-CTD using a real-time NMR deamination assay we and others have previously used for enzyme kinetics analysis of A3G-CTD^31,41^. The 9 nucleotides ssDNA 5ʹ-AATCCCAAA was used for the deamination assay, which contains the 5ʹ-TCCCA target sequence. 5ʹ-TCCCA is an optimized target sequence for A3G-CTD^41^, and Yu et al. reported that 5ʹ-CCCA is the preferential deamination sequence found in the minus strand of the HIV-1 genome^14^. Representative NMR spectra (Supplementary Fig. 1a), illustrate CTD2 deaminates the 3ʹ C in the 5ʹ-TCCCA target sequence first, then the middle C is deaminated, but the 5ʹ TC was not acted upon by CTD2 as expected based on the preference of wild-type A3G^31,41^. Indeed, the initial reaction speed was 20 times faster at pH 7.5 with 6.9 ± 0.2 reaction min^−1^ for CTD2 (red) and 0.3 ± 0.1 reaction min^−1^ for wild-type A3G-CTD (black) (Fig. 1b). This catalytic activity also increased at lower pH 6.5 (Supplementary Fig. 1b) as we previously observed for A3G-CTD^41^ with 10.8 ± 0.3 reaction min^−1^ and 0.5 ± 0.1 reaction min^−1^ for CTD2 and A3G-CTD, respectively, suggesting that CTD2 retains the wild-type catalytic mechanism while increasing the reaction speed.

Since CTD2 exhibited greater catalytic activity than A3G-CTD (Fig. 1b), we tested whether it also exhibited increased binding affinity for ssDNA to the catalytically inactive E259A variant; this construct will be referred to CTD2*. The 9 nucleotide substrate ssDNA 5ʹ-AATCCCAAA was labeled with 6-FAM modification at the 3ʹ-end for electrophoretic mobility shift assay (EMSA). Competitions with a non-specific (5ʹ-AAAAAAAAA) or a specific (5ʹ-AATCCCAAA) negative control ssDNA (fluorescent unprobed) show that CTD2* specifically binds the substrate ssDNA (Fig. 1c). The affinity of CTD2* to the 9nt substrate ssDNA was further investigated by using microscale thermophoresis (MST)^42^. The apparent dissociation constant, *K*_d_, for the CTD2* was determined to be 55 ± 12 μM (Fig. 1d). An EMSA experiment of A3G-CTD for binding the same 9nt substrate ssDNA shows significantly weaker affinity than CTD2* as only a faint shifted band observed with 160 µM of A3G-CTD (Supplementary Fig. 1d), which is consistent with the observations previously reported by other laboratories^30,31^. As expected from our previous studies^41,43^, CTD2* appeared to have significantly less affinity to 5ʹ-TCCdeoxy-UA (the first product) and 5ʹ-TCdeoxy-Udeoxy-UA (final product) because *K*_d_ values for 9nt ssDNAs containing these product sequences were determined to be 150 ± 30 μM and 5.2 ± 0.8 mM, respectively (Supplementary Fig. 1c). Furthermore, we tested CTD2* for binding the 9nt RNA containing a target sequence 5′-rArArUrCrCrCrArArA, and *K*_d_ was determined to be 1.5 ± 0.5 mM. Collectively, EMSA and MST experiments clearly indicate that CTD2* specifically binds the 5ʹ-TCCCA target DNA sequence with high affinity, making this enzyme potentially amenable for structural studies.

**Fig. 2:**
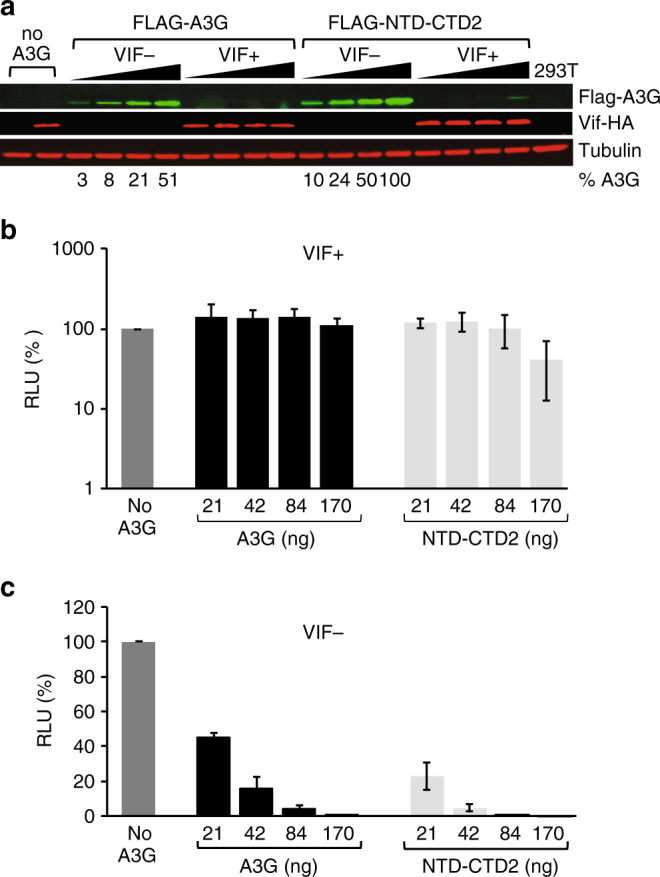
Antiviral restriction activity of FLAG-NTD-CTD2. **a** Representative western blot showing 293T cells co-transfected with increasing amounts of wild-type FLAG-A3G or FLAG- NTD-CTD2 (21, 42, 84, 170 ng; black triangles), HDV-EGFP, and VSV-G in the presence or absence of Vif-HA. Percent A3G expression is shown for each respective lane. Single-cycle infectivity of Vif+ (**b**) and Vif− (**c**) HDV-EGFP virus prepared in the presence of increasing amounts of FLAG-A3G (black bars) or FLAG-NTD-CTD2 (light gray bars) assayed in TZM-bl target cells. Data reflects the average relative light units (RLU) normalized to the no A3G control. Error bars represent the standard deviation for three independent experiments

To test whether full-length A3G containing wild-type NTD and CTD2 (NTD-CTD2) retained antiviral activity in vivo, we prepared HIV virus with increasing amounts of wild-type FLAG-A3G or FLAG-NTD-CTD2 in the presence or absence of HIV-1 Vif. Both FLAG-A3G and FLAG-NTD-CTD2 were functionally recognized by Vif and degraded as shown by the absence of A3G protein in the Vif+ lanes (Fig. 2a). As expected, neither FLAG-A3G nor FLAG-NTD-CTD2 blocked the infectivity of virus prepared in the presence of Vif in a single cycle replication assay (Fig. 2b). However, FLAG-A3G or FLAG-NTD-CTD2 potently inhibited viral infectivity when the HIV virus was prepared in the absence of Vif (Fig. 2c), and to similar extents when measuring comparable FLAG-A3G or FLAG-NTD-CTD2 protein expression levels from the producer cells (Fig. 2a) (*t*-test, *p* > 0.3). Introduction of mutation E259A into FLAG-NTD-CTD2 (and FLAG-A3G), which abolishes a critical catalytic glutamate needed for deaminase activity, restored infectivity in the absence of Vif indicating that inhibition of HIV-1 replication by FLAG-NTD-CTD2 is largely deaminase-dependent (Supplementary Fig. 2a, b and c). Furthermore, no significant differences in viral infectivities were observed for virions encapsidating similar levels of FLAG-A3G or FLAG-NTD-CTD2 (*t*-test, *p* > 0.3) (Supplementary Fig. 2d and 2e). Overall, these data confirm that the full-length NTD-CTD2 blocks HIV-1 replication as potently as wild-type A3G.

### Co-crystal Structure of CTD2* and ssDNA

**Fig. 3:**
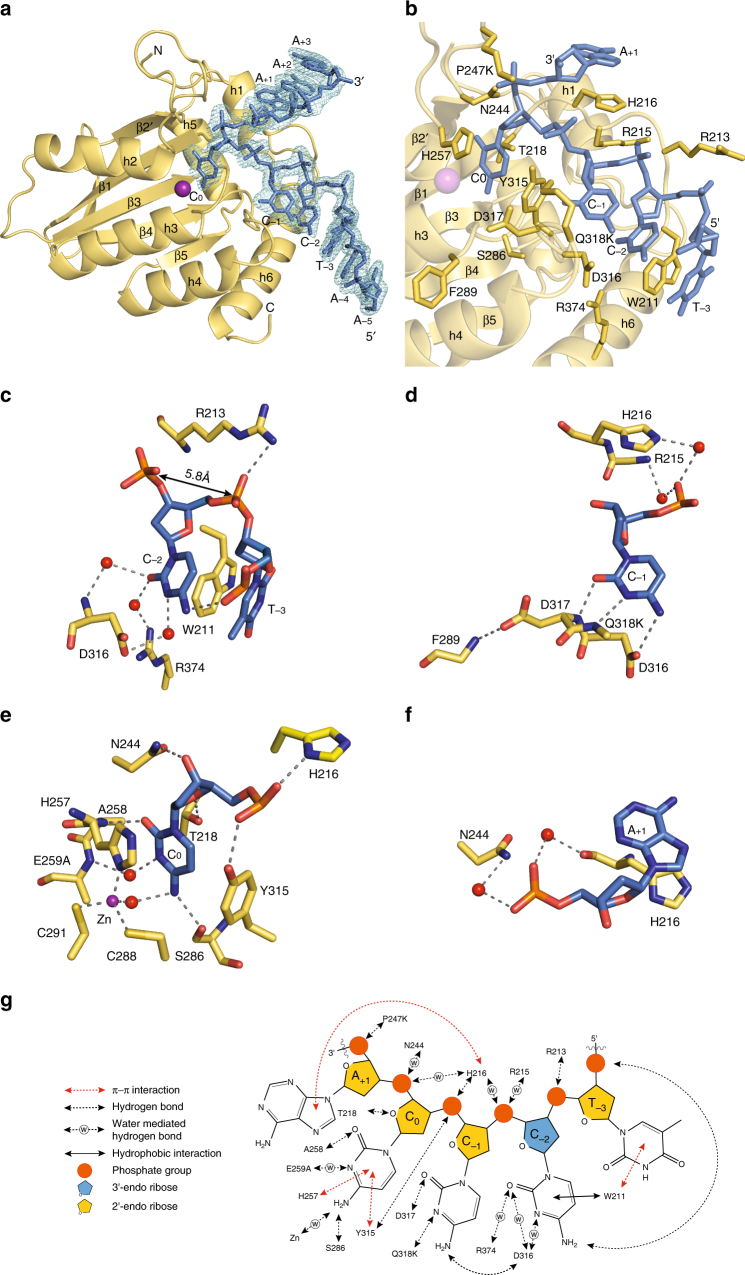
Structure of CTD2* in complex with ssDNA. **a** The asymmetric unit contains one protein (yellow) and one ssDNA (blue) molecule. A 2Fo–Fc electron density map contoured at 1*σ* is shown in cyan around the ssDNA. Zn^2+^ ion is colored purple. N and C indicate the N- and C-terminal ends of the protein, respectively. **b** An enlarged view shows interactions between the 5ʹ-TCCCA target sequence and the protein. Protein is colored yellow, 5ʹ-TCCCA is blue, and amino acid sidechains interacting with DNA are shown as sticks. **c**–**f** Enlarged views show interactions between T_−3_, C_−2_, C_−1_, C_0_, or A_+1_ and protein. C, N, and O atoms are colored yellow, blue, and red, respectively, for amino acid residues of the protein. Atoms in nucleotides are colored blue, navy blue, red, and orange for C, N, O, and P, respectively. Water molecules are shown as red spheres, and Zn^2+^ is shown as a purple sphere. Dotted lines indicate hydrogen bonds. In (**c**), the double arrow-headed line points to the neighboring backbone phosphorous atoms of C_−1_ and C_−2_. In (**d**), sidechains of F289 and Q318K are not shown. **g** Summary of the interactions between CTD2* and nucleotides in the 5ʹ-TCCCA target sequence

The catalytically inactive CTD2 (CTD2*) was co-crystallized with the 9nt ssDNA containing a 5ʹ-TCCCA target sequence (5ʹ-AATCCCAAA). The co-crystal structure of CTD2* and ssDNA was determined to 1.86 Å resolution in the P2_1_ space group by molecular replacement using our previously determined structure of apo-CTD-2K3A (PDB ID: 3IR2)^25^ (Fig. 3a). The final refinement of the structure resulted in *R*-work/*R*-free of 0.18/0.21, respectively (Table 1). There was a single CTD2*–ssDNA complex in the asymmetric unit. The overall protein backbone structure did not change significantly from the backbone structures of apo-CTD-2K3A and A3A bound to ssDNA as indicated by the pairwise root mean square (rms) deviation, which is1.6 Å for both pairs including ssDNA-bound CTD2* with apo-CTD-2K3A and DNA-bound CTD2* with ssDNA-bound A3A.

**Table 1:** Crystal data collection and refinement statistics

*Data collection*	
Space group	P2_1_
*Cell dimensions*	
a, b, c (Å)	47.41, 47.24, 51.56
α, β, γ (°)	90.00, 103.13, 90.00
Resolution (Å)	50.00–1.86 (1.93–1.86)^a^
*R*_merge_ (%)	8.3 (32.8)
*R*_meas_ (%)	8.9 (35.3)
*R*_pim_ (%)	3.3 (13.0)
I/σI	24.1 (6.3)
CC1/2	0.989 (0.957)
Completeness (%)	100.00 (100.00)
Redundancy	7.5 (7.3)
*Refinement*	
Resolution (Å)	38.6–1.86 (1.92–1.86)
No. of reflections	18,843 (1812)
*R*_work_/*R*_free_ (%)	17.89/21.11
No. of atoms	1893
Protein	1553
DNA	179
Ligand (GOL)/Ion (Zn^2+^)	6/1
Water	154
*B-factor*	
Average B-factors (Å^2^)	33.9
Protein/DNA	33.6
Ligand/ion	43.3
Water	36.6
*rms deviations*	
Bond lengths (Å)	0.006
Bond angles (°)	0.76

^a^Values in parentheses are for the highest-resolution shell

All nine nucleotides of ssDNA are well-ordered in the electron density (Fig. 3a). The interface between protein and ssDNA involves all five nucleotides of the 5′-TCCCA target sequence, and approximately 800 Å^2^ of surface area on ssDNA is buried in the interface with CTD2*. This is a significantly larger area than that found in an A3A–ssDNA complex where approximately 620 Å^2^ of surface area on ssDNA was buried in the protein–DNA interface^29^. Although more extended at both 5ʹ- and 3ʹ-ends, the phosphate backbone of the ssDNA adopted a curved shape that is similar to the shape of ssDNAs we and others observed in the co-crystal structures of A3A–ssDNA^29,39^ and TadA–RNA^44^.

Remarkably, all five nucleotides in the target sequence interact with the protein in the co-crystal structure (Fig. 3b). As others have done, in the target sequence, nucleotides are numbered with the target cytidine at position 0 such as 5′-T_−3_C_−2_C_−1_C_0_A_+1_. Therefore, we describe here protein–DNA interactions for each nucleotide in the following sections.

The most remarkable interaction involving T_−3_ is the π–π stacking with W211 (Fig. 3c). T_−3_ also interacts with the following nucleotide C_−2_ by forming a hydrogen bond between the 5ʹ-phosphate group of T_−3_ and the pyrimidine amino group of C_−2_. The Watson–Crick face of T_−3_ does not interact with the protein, whereas it forms a base pair with A_+3_ of the ssDNA in a neighboring asymmetric unit (Supplementary Fig. 3).

The nucleobase type of C_−2_ is recognized by the protein since Watson–Crick face of C_−2_ forms water-mediated hydrogen bonds between the pyrimidine carbonyl group and the mainchain amino proton of D316 as well as the guanidino group of R374 (Fig. 3c). In addition, the pyrimidine N3 atom of C_−2_ forms a hydrogen bond through an ordered water with the carboxyl group of D316 (Fig. 3c). Furthermore, the C_−2_ pyrimidine ring has a hydrophobic interaction with the indole ring of W211 (Fig. 3c), which creates a spatial restraint, favoring a pyrimidine nucleotide in this position.

Sugar pucker of nucleotides plays a key role in shaping the structure of DNA and RNA strands. The deoxy-ribose of C_−2_ has a C3ʹ-endo conformation, whereas all other eight nucleotides of the CTD2*-bound ssDNA have C2ʹ-endo conformation. This is significant because DNA prefers the C2ʹ-endo in aqueous solution, whereas RNA is predominantly in the C3ʹ-endo conformation. The C3ʹ-endo conformation of C_−2_ brings two neighboring backbone phosphorus atoms (which belong to C_−1_ and C_−2_) in a close contact 5.8 Å, which is a typical distance for double-stranded RNAs in A-form. This spatial arrangement enables the 5ʹ-phosphate group of C_−2_ to form a hydrogen bond with the guanidino group of R213. Interestingly, R213 is not conserved in other APOBEC3 proteins, except the charge is conserved with a lysine in AID, which we further describe in the Discussion.

The Watson–Crick face of C_−1_ has three direct interactions with the protein. The C2 carbonyl group forms a hydrogen bond with the mainchain amino proton of D317, the N3 atom forms a hydrogen bond with the mainchain amino proton of Q318K, and the amino group forms a hydrogen bond with the sidechain carboxyl group of D316 (Fig. 3d). The sidechain of D317 is also coordinated by a hydrogen bond formed between the carboxyl group and the mainchain amino proton of F289. This hydrogen bond stabilizes the helix3 structure by forming an “N-cap”^45^ since F289 is located at the N-terminus of the helix. In addition, Q318K may provide further support in orienting D317 by interacting electrostatically as the ε-ammonium group of Q318K is located within 3.7 Å from the carboxyl group of D317 (Supplementary Fig. 4). Furthermore, the 5ʹ-phosphate group of C_−1_ is supported by two water-mediated hydrogen bonds with the NE2 atom of H216 and the mainchain amino proton of R215 (Fig. 3d).

We observed electron density that fits a zinc ion (Zn^2+^) chelated by H257, C288, C291, and additional density that fits a water molecule. The target cytosine (C_0_) is tightly packed under the Zn^2+^ ion by stacking aromatic rings with the Zn^2+^-chelating residue H257 and forming a T-shaped π–π interaction with Y315 (Fig. 3e). In addition to these π–π interactions, many hydrogen bonds support the position of target cytosine, including aromatic ring O2 to the mainchain amino proton of A258, and aromatic ring N3 to the mainchain amino proton of E259A through an ordered water molecule. Furthermore, the deoxy-ribose O3ʹ and O4 atoms form hydrogen bonds with the sidechain amino group of N244 and the hydroxyl group of T218, respectively, which supports the 2′-endo conformation of deoxy-ribose of C_0_. The 5ʹ-phosphate group of C_0_ is well-coordinated by interactions with the protein as it forms hydrogen bonds with the hydroxyl group of Y315 and ND1 atom of H216. Two hydrogen bonds provide key recognition of the amino group of C_0_, including one formed with the mainchain carbonyl group of S286, and another formed with the water molecule coordinated by Zn^2+^. This Zn^2+^-bound water molecule is the key molecule to trigger the deamination by attacking the C4 position of cytosine^18–20^. These C_0_-interacting residues are conserved in all APOBEC3 proteins (except A3F has a serine instead of T218), and we and others have observed similar interactions in A3A–ssDNA complexes^29,39^. Although the CTD2*–ssDNA complex showed that C2ʹ-endo sugar conformation of the target cytidine is required to fit in the catalytic pocket, the mechanism by which A3G discriminates RNA from deamination is not fully understood because the 2ʹ hydrogen of the target cytidine can be replaced with a hydroxyl group without significant steric hindrance in the CTD2*–ssDNA complex structure.

The purine ring of A_+1_ stacks against the H216 imidazole ring (Fig. 3f). Since histidine forms stronger π–π stacking with a purine ring than with a pyrimidine ring^46^, this interaction selects purines in the +1 position rather than pyrimidines, providing an explanation for why 5ʹ-CCCA is the preferential deamination sequence^14,41^. In addition, the 5ʹ-phosphate group forms water-mediated hydrogen bonds with the mainchain carbonyl group of H216 and the sidechain amino group of N244. The Watson–Crick face of A_+1_ does not have interaction with protein as it forms a base pair with A_−5_ of a neighboring asymmetric unit (Supplementary Fig. 3).

Overall, CTD2* recognizes 5ʹ-C_−2_C_−1_C_0_ through hydrogen-bonds formed with their Watson–Crick faces, and T_−3_ and A_+1_ by using strong π–π interactions (Fig. 3g). Unusual sugar pucker of C_−2_ contributes in shaping the phosphate backbone to fit the ssDNA-binding site of CTD2*. The movements of CTD2* residues induced upon the ssDNA-binding will be discussed in the following section.

## Discussion

Since we reported the first structure of the catalytic domain of A3G almost 10 years ago^23^, structural studies of the A3G-CTD–ssDNA complex have been hampered by apparently weak binding of A3G-CTD to ssDNA^23,31^. We have overcome this problem by generating a A3G-CTD variant that binds ssDNA with higher affinity than wild-type. P247K appears to be a key substitution that contributes to stabilizing the ssDNA-binding of CTD2* by providing an additional hydrogen bond to a backbone phosphate group located outside of the target sequence (Supplementary Fig. 4). Furthermore, non-Watson–Crick base pairs formed between neighboring asymmetric units in the CTD2*–ssDNA co-crystal may stabilize the crystallization of the complex (Supplementary Fig. 3). Critically, this highly active variant in the context of full-length A3G containing wild-type NTD restricted HIV-1 infection as potently as wild-type A3G in a Vif-dependent manner (Fig. 2).

**Fig. 4:**
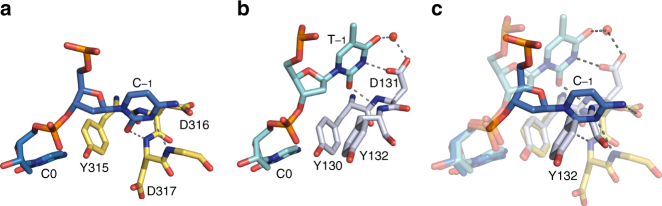
Comparison of CTD2* and A3A recognition of the nucleotide at −1 position. **a** Interaction of cytidine at −1 position (C_−1_, blue) with residues of CTD2* (yellow) in the CTD2*–ssDNA complex (this study). **b** Interaction of thymidine at −1 position (T_−1_, cyan) with residues of A3A (gray) in the A3A–ssDNA complex (PDB ID: 5KEG). **c** Superimposition of the CTD2*–ssDNA and A3A–ssDNA complexes showing nucleotides at the −1 position and their interacting residues from both structures

A3G strongly prefers a cytidine at the −1 position, whereas A3A prefers a thymidine for that position. CTD2 and A3A form hydrogen bonds with the Watson–Crick face of the nucleotide at the −1 position to provide nucleobase specificity. For the CTD2*–ssDNA complex, the Watson–Crick face of C_−1_ forms three hydrogen bonds including amino group (NH_2_) to carboxyl group of D316, carbonyl group to mainchain amino proton of D317, and N3 atom to mainchain amino proton of Q318K. If this cytidine is replaced by a thymidine, N3 and NH_2_ would be replaced by NH and CO, respectively, resulting in the loss of two hydrogen bonds with the protein, which explains why A3G prefers cytidine over thymidine at the −1 position. Previously, Rausch et al. showed that N3 and NH_2_ of cytosine ring of C_−1_ and C_−2_ are key for deamination efficiency, whereas 5-methyl deoxy-cytidine at C_−1_ or C_−2_ position are tolerated by A3G^47^. This finding is consistent with the CTD2*–ssDNA structure showing that Watson–Crick faces of C_−1_ and C_−2_ interact with CTD2*, while the C5 positions do not have a contact with the protein (Fig. 3b). In addition to the recognition of the Watson–Crick face, the spatial coordination plays an important role in the recognition of C_−1_. Figure 4a, b shows a striking difference between the position of C_−1_ in the CTD2*–ssDNA complex and that of T_−1_ in the A3A–ssDNA complex. Interactions of T_−3_ and C_−2_ with CTD2* are important to position C_−1_ (Fig. 3b, c), whereas the A3A–ssDNA structures did not have interactions with nucleotides at −2 and −3 positions^29,39^. A3A has a tyrosine (Y132) at the corresponding position to D317, and Fig. 4c shows that C_−1_ crashes into Y132 when we overlay the CTD2*–ssDNA structure onto the A3A–ssDNA structure. The significance of D316 and D317 in substrate specificity of A3G was originally reported by Holden and co-workers as they showed that D316R, D317R double substitutions enabled A3G to deaminate middle C and 3′ C of a 5ʹ-CCC motif at the same reaction speed^30^, whereas the wildtype A3G-CTD prefers 3ʹ C to the middle C by 45-fold^41^. Another group showed that D317Y substitution changed A3G substrate preference to 5′-TC that is the A3A preferred target sequence^48^. The co-crystal structure of CTD2* and ssDNA showed how D316 and D317 are involved in the recognition of C_−1_. Overall, spatial coordination as well as the interaction with the Watson–Crick face are critical for specific recognition of C_−1_.

**Fig. 5:**
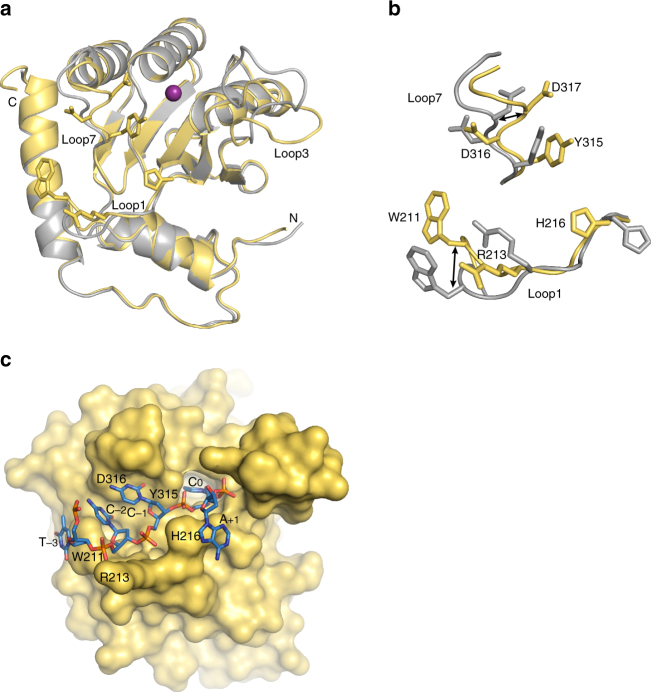
Comparison of structures of CTD2* with apo-CTD-2K3A. Proteins are in the same orientation in all three figures. **a** Superimposed yellow and gray cartoons show structural features of CTD2* (this study) and CTD-2K3A (PDB ID# 3IR2), respectively. W211, R213, H216, Y315, D316, and D317 sidechains of CTD2* are shown in sticks. Zn^2+^ ion is shown as a purple sphere. ssDNA is not shown. **b** An enlarged view of (**a**) that shows the repositioning of the critical residues W211, R213, and H216 of loop1, and Y315, D316, and D317 of loop7. Double headed arrows point to positions of Cα atoms of W211 and D317. **c** Surface representation of ssDNA-bound CTD2* (this study). Locations of the loop1 and loop7 residues are labeled except D317 because D317 is not seen on the surface. The 5ʹ-TCCCA target sequence is shown as sticks, and C, N, O, and P atoms are colored blue, dark blue, red, and orange, respectively

We and others have reported structures of A3G-CTD wild-type^30,31,35^, and a soluble variant, namely CTD-2K3A^23,25^. None of these structures were complexed with ssDNA, and we chose our previous crystal structure of CTD-2K3A, solved at 2.25 Å resolution (PDB ID# 3IR2^25^), as a representative of apo-form CTD for structural comparison with ssDNA-bound CTD2*. Superimposition of structures of ssDNA-bound CTD2* (yellow) and apo-CTD-2K3A (gray) (Fig. 5a) reveals that even with the additional substitutions in CTD2* the backbone structures are essentially unchanged. Regions that are changed include loops 1, 3, and 7, which are intrinsically dynamic in solution as shown in NMR structures of CTD-2K3A^23,24^. Loops 1 and 7 contain amino acid residues, which form numerous interactions with ssDNA; therefore, the structural changes were likely induced upon ssDNA binding.

Loop1 migrates toward loop7 upon ssDNA binding, with W211 demonstrating the biggest change with its Cα atom moved by 3.9 Å from the position found in the apo-form CTD-2K3A (Fig. 5b). This backbone change enables W211 to have π–π stacking interaction with T_−3_. The sidechain of H216 showed a big rotamer change, enabling π–π stacking with A_+1_ (Fig. 5b). These π–π interactions set both 5ʹ- and 3ʹ-ends of the 5ʹ-TCCCA target sequence to the rim of the DNA-binding groove formed by loop1 of CTD2*. This rim is clearly visible by sidechains of W211, R213, and H216 in the surface representation of CTD2* (Fig. 5c). This loop1 rim interacts with the phosphate backbone of ssDNA, whereas loop7 faces nucleobases as depicted by the sidechains of Y315 and D316 (Fig. 5c). Residues in loop7, including Y315, D316, and D317, also alter mainchain atom positions and sidechain rotamers upon ssDNA-binding (Fig. 5b). D317 significantly changed its mainchain position as its Cα atom moved 3.0 Å from the position found in the apo-form CTD-2K3A structure (Fig. 5b). These rearrangements of backbone atoms of the loop7 residues are particularly important for recognition of C_−1_, as mainchain amino protons form two hydrogen bonds with the Watson–Crick face of C_−1_ (Fig. 3d). This dynamic property of CTD2* is a remarkable difference compared to A3A, which did not show significant changes in mainchain atom positions upon ssDNA-binding. For A3A, only the sidechains of R28 and H29 (R215 and H216 in CTD2, respectively) in loop1, and Y132 (Y315 in CTD2) in loop7 changes their rotamers^29,39^.

Most recently, Ziegler et al. published a structure of A3G-CTD bound to an adenine nucleotide^49^. In the A3G-CTD–adenine complex structure, the adenine nucleotide binds in the space that is similar to the T_−1_ position found in the A3A–ssDNA complexes^29,39^. Since C_−1_ in the CTD2*–ssDNA complex does not occupy the T_−1_ position (Fig. 4c), the protein–DNA interaction found in the A3G-CTD–adenine complex^49^ is different from the enzyme–substrate interaction revealed in our study. Ziegler et al. suggested that the A3G-CTD–adenine structure shows a non-specific interaction by which A3G-CTD scans ssDNA sequence^49^. Interestingly, W211 rearranged its position to interact with the bound adenine^49^, which may imply that W211 is important in the interaction with non-specific DNA as well as the target sequence.

As mentioned in the previous section, P247K is the only residue in loop3, which interacts with ssDNA, and the interaction likely contributes in changing the loop3 position (Supplementary Fig. 4a). In addition, other loop3 residues including E254 and R256 form hydrogen bonds with Q293 (helix3) and E323 (helix4) of a neighboring asymmetric unit, respectively, which support the position of loop3 and concurrently aids crystal formation. Noteworthy, the substitutions introduced to CTD2 did not change the structure of A3G-CTD as shown in the superimposed structures of CTD2* (this study) and wild-type A3G-CTD (PDB ID: 4ROV)^35^ (Supplementary Fig. 4b). CTD2* and 4ROV structures are well superimposed as indicated by the pairwise rms deviation, which is 0.9 Å.

We have previously found that A3G-CTD increases its catalytic activity at lower pH, and H216 plays a key role in this pH dependence^41^. Alanine mutation of this residue abolished catalytic activity, whereas arginine mutation kept catalytic activity but lost the pH dependency, suggesting that positive charge and/or formation of hydrogen bonds of this residue are important for substrate binding^41^. The CTD2*–ssDNA co-crystal structure indicates that protonation of the imidazole ring enables the formation of two hydrogen bonds with the 5ʹ-phosphate groups of C_0_ and C_−1_. H216 is conserved only in A3G and A3A among human APOBEC3 proteins, and similar interactions between the histidine and nucleotides at −1 (T_−1_) and 0 (C_0_) positions have been observed in co-crystal structures of A3A and ssDNA^29,39^. These conserved interactions involving histidines provide an explanation for similar pH dependency of catalytic speeds of A3A and A3G^41,50^.

Until this study, we have been puzzled how W211 contributes to catalysis as this residue is spatially far from the catalytic Zn^2+^ ion yet alanine substitution of W211 results in nearly complete loss of catalytic activity^23^. Moreover, we observe large chemical shift changes for the indole amino proton of W211 upon mixing with ssDNAs^23,51^. Interestingly, while W211 is not conserved in other members of human APOBEC3 proteins, tryptophan is conserved in AID. AID is a member of the human APOBEC family, that plays important roles in antibody diversification and triggers both class switch recombination and somatic hypermutation^52–54^. Qiao et al. recently published structures of the human AID protein co-crystalized with dCMP, cacodylic acid or cytidine (PDB ID# 5W0U, 5W0R, 5W1C, respectively), and proposed a “substrate channel” composed by loop1 and loop7 of AID^40^. AID appears to recognize long target sequences, similar to A3G recognition of a five nucleotide target sequence, and AID has nucleotide preferences in −2, −1, 0, and +1 positions^55,56^. Based on the CTD2*–ssDNA complex, AID likely uses the tryptophan residue corresponding to W211 for π–π stacking with the nucleotide at the −3 position, and supports specific recognition of the nucleotides at −2 and −1 positions in a manner similar to CTD2* use of W211. In addition, conservation of R213 of CTD2* as a lysine in AID suggests the similar use of the lysine for interaction with 5ʹ-phosphate group of the nucleotide at −2 position. Further experimental data and structures are necessary to elucidate the ssDNA interactions of AID.

This structure of the CTD2*–ssDNA complex reveals the mechanism at atomic-level resolution by which the catalytic domain of A3G uniquely binds substrate ssDNA. Fundamental knowledge of this complex can guide the design of molecular-based therapeutics for AIDS by modulating A3G catalytic function. In addition, recently, Komor et al. showed that an APOBEC deaminase tethered to catalytically dead Cas9 can mutate DNA in a programmable manner, offering a new strategy for “gene editing”^57^. The CTD2 variant may be a better tool for “base editing” because it is more soluble, binds ssDNA stronger and catalyzes deamination faster than wild-type A3G-CTD.

## Methods

### Protein Expression and Purification

The CTD2 variant of human A3G CTD (residue 191–384) and its inactive variant CTD2* were expressed from pGEX6P-1 expression plasmid [P247K was introduced by synthesizing cDNA (GenScript), and the primer sequences used for E259A and Q318K substitutions are listed in Supplementary Table 1] with Glutathione S-transferase (GST) tag (crystallography, EMSA, and real-time NMR deamination assay) for GST purification or from pET-28a plasmid with poly-Histidine tag (MST assay) for Ni-NTA purification in *E. coli* BL21(DE3) cells (Invitrogen). Cells were grown in LB media at 37 °C until reaching an optical density of 0.5–0.6 at 600 nm. Then, temperature was reduced to 17 °C and protein expression was induced for 18 h with 0.2 mM isopropyl β-D-1-thiogalactopyranoside (IPTG).

All the steps for protein purification were performed at 4 °C. *E. coli* cells were harvested by centrifugation and re-suspended in lysis buffer (either 50 mM sodium phosphate pH 7.3, 150 mM NaCl, 25 µM ZnCl_2_, 2 mM DTT, and 0.002% Tween-20 for GST purification or 50 mM sodium phosphate pH 7.3, 150 mM NaCl, 50 μM ZnCl_2_, 1 mM DTT, and 0.002% Tween-20 for Ni-NTA purification) and EDTA-free protease inhibitor cocktail (Roche, Basel, Switzerland). The suspended cells were disrupted by sonication and then cell debris were separated by centrifugation at 48,384*g* for 30 min. Supernatant containing desired protein was applied to either Glutathione-Sepharose resin (GE Healthcare Life Science) for GST purification or Ni-NTA Agarose resin (QIAGEN) for Ni-NTA purification, equilibrated with lysis buffer and agitated for about 2 h.

For GST purification, protein-bound resin was washed with Pre-Scission Protease cleavage buffer (50 mM sodium phosphate, pH 7.5, 100 mM NaCl, 10 µM ZnCl_2_, 2 mM DTT, and 0.002% Tween-20) and incubated with Pre-Scission protease (GE Healthcare Life Science) for 18 h. The supernatant containing the cleaved protein was separated from the resin by centrifugation and loaded on to HiLoad 16/600 Superdex 75 gel filtration column (GE Healthcare Life Science) equilibrated with 20 mM Bis–Tris (pH 6.5), 100 mM NaCl, 1 mM DTT, 0.01 mM ZnCl_2_, and 0.002% Tween-20.

For Ni-NTA purification, protein-bound resin was washed with 50 mM sodium phosphate, pH 7.3, 1 M NaCl, 25 µM ZnCl_2_, 1 or 2 mM DTT, and 0.002% Tween-20. Protein was eluted from resin in buffer containing 400 mM imidazole, 50 mM sodium phosphate, pH 7.3, 100 mM NaCl, 1 mM DTT, and 0.002% Tween-20. Eluted protein was loaded on to HiLoad 16/600 Superdex 75 gel filtration column equilibrated with 20 mM Bis–Tris pH 6.5, 100 mM NaCl, 1 mM DTT, 0.002% Tween-20, and 20 μM ZnCl_2_. For both GST and Ni-NTA purification, protein purity was analyzed by SDS-PAGE.

### Crystal Growth and Data Collection

Samples used for crystallization contained about 9.5 mg/ml (415 µM) CTD2* and a 50% molar excess of ssDNA in 20 mM Bis–Tris pH 6.5, 100 mM NaCl, 1 mM DTT, 10 µM ZnCl_2_, and 0.002% Tween-20. The 9 nucleotide ssDNA, 5ʹ-AATCCCAAA, was obtained from Integrated DNA Technologies (IDT; Coralville, IA). Initial crystallization condition was identified using JBScrene Nuc-Pro from MiTeGen. Crystals were grown at 4 °C, by sitting drop vapor diffusion method over a 65 µl reservoir of 20% W/V PEG 6000, 50 mM di-sodium L-malate; pH 5.0 and 30 mM CaCl_2_ in a sitting drop 2 well crystallization plate from Molecular Dimension. Drops were set up by mixing 0.3 µl of CTD2*–ssDNA complex and 0.3 µl of reservoir solution using a robot, Mosquito Crystal from TTP Labtech. Crystals appeared after 1 week. Crystals grown at 4 °C were melted at room temperature, and exactly similar crystal setup at 20 °C did not produce any crystal.

Crystals were cryoprotected using reservoir solution containing 20% v/v glycerol and flash frozen in liquid nitrogen. X-ray diffraction data were collected at Southeast Regional Collaborative Access Team (SER-CAT) 22-ID beamline at the Advanced Photon Source, Argonne National Laboratory. The crystals belong to the space group P2_1_. The collected intensities were indexed, integrated, and scaled using HKL2000^58^.

### Structure Determination and Analysis

The structure was solved at 1.86 Å resolution by molecular replacement using the program Phaser^59^ and a previously determined structure of A3G-2K3A (PDB ID code 3IR2, chain B was removed) as search model^25^. Model building of the protein and bound DNA and refinements were manually performed using the programs Coot^60^ and Phenix^61,62^, respectively. The first 3 residues (Glu–Ile–Leu) and the last residue (Asn) were not modeled due to lack of electron density. Due to the presence of extra positive density, Ser-368 and Ser-372 were modeled in two alternative conformations. The final model was refined to *R*_work_/*R*_free_ values of 0.18/0.21 and was validated with the PDB validation tool and Molprobity^63^. Statistics of Ramachandran analysis yielded 97.87% of the residues in the favored regions and 2.13% were found in the allowed regions. None of the residues were found in disallowed regions. Pairwise rms deviation between CTD-2K3A and ssDNA-bound CTD2* and between ssDNA-bound A3A and ssDNA-bound CTD2* were calculated using Doli^64^. Figures of structure models were generated by PyMOL^65^.

### Real-time NMR Deamination Assay

We determined initial rates of deamination reaction by using ^1^H NMR spectra. A 9nt ssDNA substrate (IDT), 5ʹ-AATCCCAAA, was used to determine the reaction rate. NMR spectra were acquired at 25 °C on Bruker NMR spectrometers operating at ^1^H Larmor frequencies of 600 and 800 MHz. NMR samples contained 5% deuterium oxide with 200 nM protein, 200 µM ssDNA substrate, 100 mM NaCl, 0.002% Tween 20, 1 mM DTT, 10 µM ZnCl_2_, and also included 50 mM sodium phosphate adjusted to pH 7.5. The concentration of deamination product (5ʹ-AATCCdeoxy-UAAA) was determined from integration of the H5 uracil proton peaks at 5.60 ppm. A series of ^1^H spectra were measured and the product concentrations as a function of the reaction times were used to determine the initial rate via linear regression.

### EMSA

For EMSA, we used a 9nt ssDNA with 6-FAM at the 3ʹ end 5ʹ-AATCCCAAA-FAM (IDT), and binding buffer containing 20 mM Bis–Tris pH 6.5, 100 mM NaCl, 1 mM DTT, 10 µM ZnCl_2_, and 0.002% Tween 20 detergent. To test the specific DNA binding, we performed competition assays with fluorescent-unprobed ssDNAs including 5ʹ-AAAAAAAAA and 5ʹ-AATCCCAAA, as a non-specific and a specific ssDNA, respectively. Binding reactions were performed in 50 µl by mixing the 6-FAM-labeled ssDNA (5ʹ-AATCCCAAA-FAM) with CTD2* at 10 nM and 15 μM concentration, respectively. A competitor ssDNA was added with incremental amounts, 0, 25, 250, and 2500 nM. Reaction mixtures were incubated for 1 h at room temperature. Samples (10 μl) were mixed with Novex Hi-Density TBE sample buffer (5× loading dye from Invitrogen) and loaded onto a 4–12% precast TBE gel (Invitrogen) and run with 0.5× TBE buffer for 60 min at 100 V at 4 °C. Gels were imaged by using a Typhoon imager (GE Healthcare Life Sciences) using the blue-excitation (488 nm) fluorescence mode.

### Microscale Thermophoresis Assay

The binding affinity of purified CTD2* to 9nt ssDNA 5ʹ-AATCCCAAA (IDT) was measured using Monolith NT.115 (Nano Temper Technologies, GmbH, Munich, Germany)^42^. RED-tris-NTA fluorescent dye solution was prepared at 100 nM in the MST buffer (20 mM Bis–Tris pH 6.5, 100 mM NaCl, 1 mM DTT, 0.002% Tween 20, 20 μM ZnCl_2_). CTD2* was mixed with dye at final concentration of 100 nM and incubated for 30 min at room temperature followed by centrifugation at 15,000*g* for 10 min. The ssDNA was prepared to a stock concentration of 8 mM in the MST buffer. To determine the binding affinity, 10 μl of ssDNA solution at 16 different concentrations, ranging from 8 mM to 0.244 µM, were prepared in LoBind centrifuge tubes (Fisher Scientific), then 10 μl of fluorescent-labeled CTD2* solution was added to each tube. The mixtures were incubated at 4 ^°^C to reach equilibrium. Each incubated solution was loaded into a Nano Temper MST premium coated capillary. The measurement was performed at room temperature using 40% LED power and 20% MST power. The experiment was repeated three times and data analysis was carried out using Nano Temper analysis software (MO affinity).

### HIV-1 Restriction Assay

The plasmids in this study are designated with a “p” while the names of viruses and proviruses generated from these plasmids are not. pHCMV-G expresses the G glycoprotein of vesicular stomatitis virus (VSV-G)^66^. pHDV-EGFP is an HIV-1 derived vector that expresses HIV-1 Gag-Pol and enhanced green fluorescent protein (EGFP) but does not express Env, Vif, Vpr, Vpu, or Nef^67^. pVif-HA is a codon-optimized HIV-1 Vif expressing a C-terminal HA epitope tag^68^. pFLAG-A3G expresses wild-type A3G with an N-terminal FLAG epitope tag^69^. To create pFLAG-NTD-CTD2, unique EcoRI and XbaI cloning sites from pFLAG-A3G were used to subclone a codon-optimized A3G containing solubility mutations 2K3A (L234K + C243A + F310K + C321A + C356A)^23^ and mutations P199A, P200A, N236A, P247K, Q318K, and Q322A. To create pFLAG-A3G-E259A and pFLAG-NTD-CTD2-E259A, plasmids pFLAG-A3G and pFLAG-NTD-CTD2 were subjected to site-directed mutagenesis to introduce E259A (Quick Change Lightning Site-Directed Mutagenesis Kit, Agilent Technologies). All final plasmids were confirmed by sequencing (Macrogen).

Human embryonic kidney 293T cells (American Type Culture Collection) and TZM-bl cells (obtained through the NIH AIDS Reagent Program [Cat. No. 8129], Division of AIDS, NIAID, NIH: TZM-bl from Dr. John C. Kappes, Dr. Xiaoyun Wu, and Tranzyme Inc.^70^) were grown in Dulbecco’s modified Eagle’s medium (DMEM) supplemented with 10% fetal calf serum (HyClone) and 1% penicillin–streptomycin stock (penicillin 50 U/ml and streptomycin 50 μg/ml, final concentration; Gibco). TZM-bl cells contain a HIV-1 tat-inducible luciferase reporter gene that correlates with HIV-1 infectivity. All cells were maintained in humidified 37 °C incubators with 5% CO_2_.

All transfections were performed using LT1 reagent (Mirus) according to manufacturer’s instructions. To generate virus for infection, in brief, 293T cells were transfected with pHDV-EGFP (1 μg), with or without pVif-HA (2.5 μg), pHCMV-G (0.25 μg), and variable concentrations of pFLAG-A3G or pFLAG-NTD-CTD2 (21, 42, 84, 170, or 340 ng). To maintain equivalent DNA amounts in the transfection mix, pcDNA3.1 was substituted as needed. Forty-eight hours post infection, virus was harvested, filtered with 0.45-μM filters, and stored at −80 °C. Capsid p24 measurements were analyzed using the HIV-1 p24 ELISA Kit (XpressBio) according to manufacturer’s instructions. Normalized p24 was used to infect 4000 TZM-bl cells in a 96-well plate, and 48 h post infection, luciferase activity was measured using a 96-well luminometer (LUMIstar Galaxy, BMG LABTECH). Data were plotted as the percent inhibition of luciferase activity versus the no A3G control. For some experiments, portions of the viral supernatant were spun through a 20% sucrose cushion (15,000 rpm, 2 h, 4 °C, in a Sorvall WX80 + ultracentrifuge) and concentrated 10-fold and used in experiments to determine virion encapsidation of FLAG-A3G and FLAG-NTD-CTD2 by western blotting analysis.

Cell lysates were made using CelLytic M (Sigma) solution containing Protease Inhibitor Cocktail (Roche), followed by a 10 min, 10,000×*g* spin to remove cellular debris. The cell lysates were mixed with NuPAGE LDS sample buffer (Invitrogen) containing β-mercaptoethanol and heated for 5 min at 95 °C. Samples were analyzed on 4–20% Tris-Glycine Gels (Invitrogen) using standard western blotting techniques. Proteins were detected with primary antibodies as follows: FLAG-A3G or FLAG-NTD-CTD2 (rabbit anti-FLAG polyclonal antibody, 1:5000 dilution, Sigma #F7425); Vif-HA (mouse anti-HA monoclonal antibody, 1:5000 dilution, Sigma #H3663); α-tubulin (mouse anti-α-tubulin antibody, 1:10,000 dilution, Sigma #T9026); Antibody against HIV-1 p24 (monoclonal, 1:10,000 dilution) was obtained through the NIH AIDS Reagent Program, Division of AIDS, NIAID, NIH: HIV-1 p24 Gag Monoclonal (#24-3) from Dr. Michael Malim (catalog #6458)^71^. An IRDye 800CW-labeled goat anti-rabbit secondary antibody (LI-COR #926-32211) was used at a 1:10,000 dilution to detect rabbit primary antibodies and an IRDye 680-labeled goat anti-mouse secondary antibody (LI-COR #926-68070) was used at a 1:10,000 dilution to detect mouse primary antibodies. Protein bands were visualized and quantified using an Odyssey Infrared Imaging System (LI-COR). Uncropped western blots are shown in Supplementary Figs. 6, 7, and 8.

### Data Availability

Atomic coordinates and structural factors for the reported crystal structure have been deposited in the Protein Data Bank under the accession number 6BUX. Other data are available from the corresponding author upon reasonable request.

## Electronic supplementary material

Supplementary Information

Peer Review Report
