# Supplementary material for: Crystal structure of the catalytic domain of HIV-1 restriction factor APOBEC3G in complex with ssDNA
Source: Nat Commun. 2018 Jun 25;9:2460. doi: 10.1038/s41467-018-04872-8 (PMC6018426; doi:10.1038/s41467-018-04872-8)
Supplement: Supplementary file 1 — Supplementary Information [file 41467_2018_4872_MOESM1_ESM.pdf]

Supplementary Information

Crystal structure of the catalytic domain of HIV-1 restriction factor APOBEC3G in complex with ssDNA

Maiti et al.

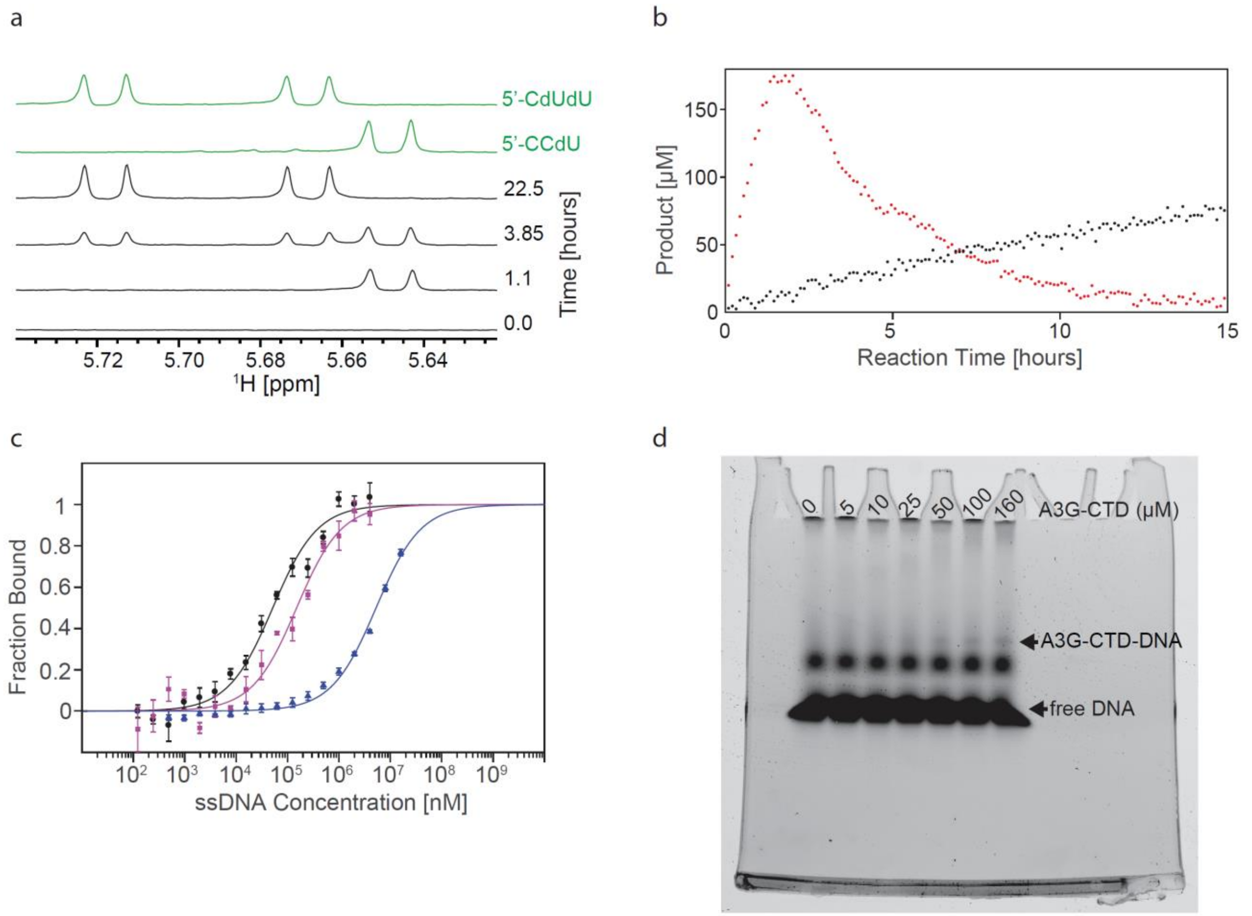

Supplementary Figure 1.

Real-time NMR deamination assay and ssDNA binding of CTD2. (a) An enlarged region of a ^{1}H spectrum of the substrate 5′-AATCCCAAA is compared at several time points during deamination reactions to illustrate the increase in the H5 proton signal of uracil due to the deamination of the 3′ C (a doublet signal centered at 5.65ppm at pH 6.5), produced 5′-AATCCdeoxy-UAAA, followed by the deamination of the central C (a doublet signal centered at 5.72ppm at pH 6.5) produced 5′-AATCdeoxy-Udeoxy-UAAA. Spectra of 5′-AATCCdeoxy-UAAA and 5′-AATCdeoxy-Udeoxy-UAAA are provided as references (green spectra labeled 5′-CCdU and 5′-CdUdU, respectively). Due to the change in chemical environment, after formation

of the second deoxy-U, the position of the first deoxy-U H5 proton signal shifts from 5.65 ppm to 5.67ppm.

(b) The first deamination product (5′-AATCCdeoxy-UAAA) concentration as a function of reaction time is plotted for CTD2 (red dots) and wild-type A3G-CTD (black dots) deamination at pH 6.5 with 200nM protein and 200µM 5′-AATCCCAAA substrate. For CTD2, the first deamination approached completion within ~1.5 hours, then the second deamination (5′-AATCCdeoxy-UAAA to 5′-AATCdeoxy-Udeoxy-UAAA) started, which decreased the first product (5′-AATCCdeoxy-UAAA) concentration. (c) Each dot represents microscale thermophoresis (MST) measurement of a mixture containing fluorescent-labeled CTD2* (50nM) and 9nt ssDNAs including 5′-AATCCCAAA (black), 5′-AATCCdeoxy-UAAA (magenta) and 5′-AATCdeoxy-Udeoxy-UAAA (blue) at various concentrations including 0.12 µM, 0.24 µM, 0.48 µM, 0.97 µM, 1.95 µM, 3.90 µM, 7.81 µM, 15.62 µM, 31.25 µM, 62.5 µM, 125 µM, 250 µM, 500 µM, 1 mM, 2 mM and 4 mM. Additionally, we measured MST at 8mM and 16mM concentration for 5′-AATCdeoxy-Udeoxy-UAAA (blue). Three independent MST experiments were performed, and bars of data points represent standard error of n=3 measurements. (d) EMSA for binding of wild-type A3G-CTD* (catalytically inactive) to the 9nt ssDNA 5′-AATCCCAAA-6-FAM. A3G-CTD-DNA indicates position of the A3G-CTD*-ssDNA complex, and free DNA indicates the position of protein-free ssDNA. 10 nM 5′-AATCCCAAA-6-FAM was incubated with incremental amounts of A3G-CTD*, including 0, 5, 10, 10, 25, 50, 100 and 160 µM in lanes 1, 2, 3, 4, 5, 6 and 7, respectively.

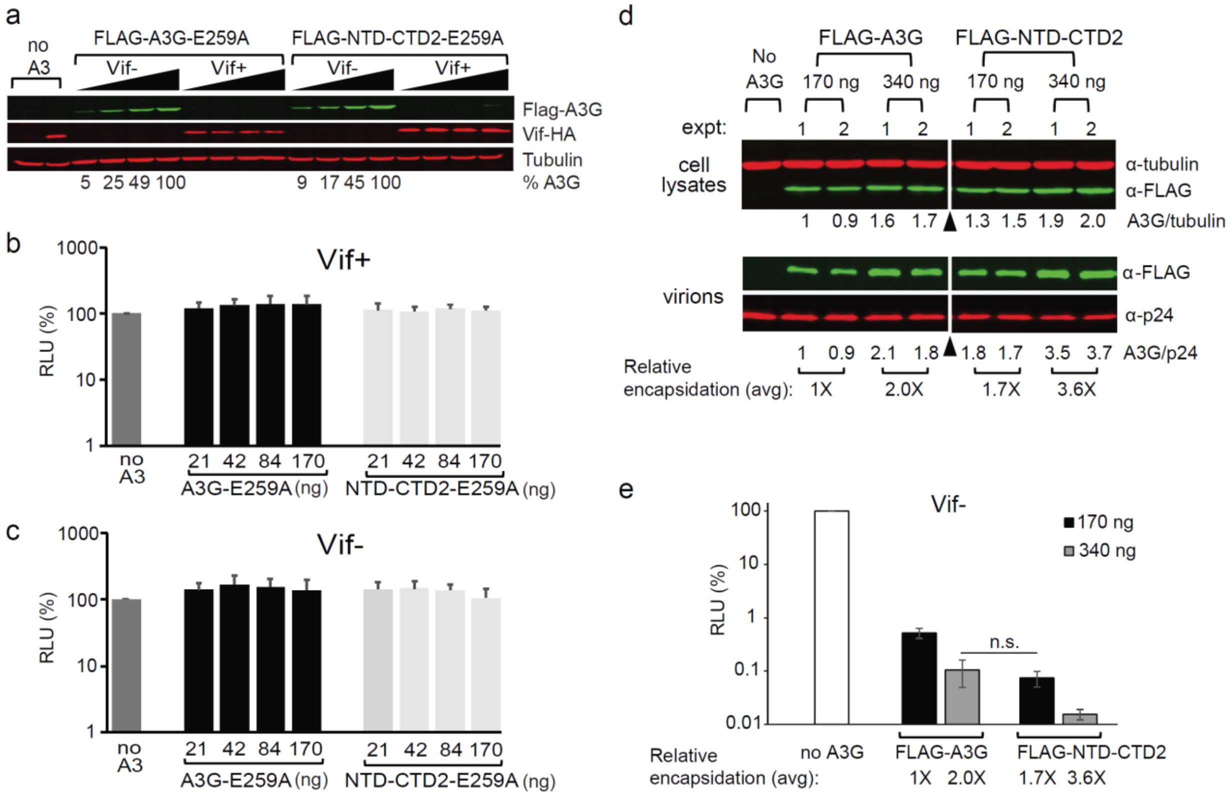

Supplementary Figure 2.

Antiviral activity of FLAG-A3G-CTD2 is dependent on its deaminase activity, and viral encapsidation of FLAG-A3G and FLAG-NTD-CTD2 correlates with infectivity. (a) Representative western blot showing 293T cells co-transfected with increasing amounts of wild-type FLAG-A3G-E259A or FLAG-NTD-CTD2-E259A (21, 42, 84, 170 ng; black triangles), HDV-EGFP and VSV-G in the presence or absence of Vif-HA. Percent A3G expression is shown for each respective lane. Absence of FLAG signal in the Vif+ lanes indicates that the E259A mutants were efficiently degraded by Vif. Single-cycle infectivity of Vif+ (b) and Vif- (c) HDV-EGFP virus prepared in the presence of increasing amounts of FLAG-A3G-E259A or FLAG-NTD-CTD2-E259A assayed in TZM-bl target cells. Data reflects the average relative light units (RLU) normalized to the no A3G control. Error bars represent the standard deviation for eight

independent infections from two viral preps. (d) Representative western blot of 293T cell lysates and virions produced from 293T cells co-transfected with FLAG-A3G or FLAG-NTD-CTD2 (170 ng or 340 ng), HDV-EGFP and VSV-G in the absence of Vif-HA. FLAG-A3G or FLAG-NTD-CTD2 expression was normalized to tubulin levels in cell lysates and capsid p24 levels in virions. Fold change in expression levels is shown below each respective lane normalized to FLAG-A3G (170 ng, Expt 1). (e) Single-cycle infectivity of HDV-EGFP virus prepared in the presence of increasing amounts of FLAG-A3G or FLAG-NTD-CTD2 assayed in TZM-bl target cells. Data reflects the average relative light units (RLU) normalized to the no A3G control. Error bars represent the standard deviation for four independent infections from two viral preps. n.s., not significant (*t*-test, $p > 0.3$). Black triangle, lanes derived from the same blot with extraneous lanes removed.

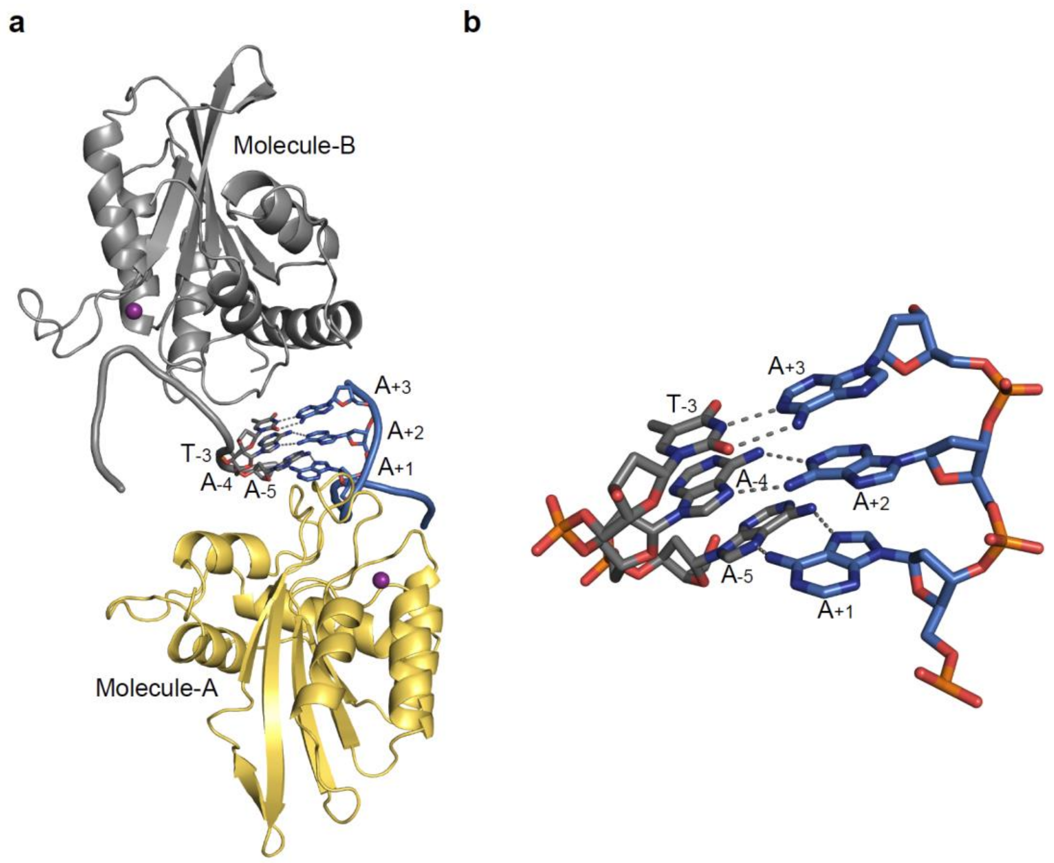

Supplementary Figure 3.

Crystal contacts: base pairs formed between two asymmetric units. There are three base pairs formed between two neighboring asymmetric units, which involve 5′-$A_{-5}A_{-4}T_{-3}$ from one unit and 5′-$A_{+1}A_{+2}A_{+3}$ from the other unit. In these inter-molecular base pairs, 5′-$A_{-5}A_{-4}T_{-3}$ and 5′-$A_{+1}A_{+2}A_{+3}$ strands are oriented in parallel, and form A_{-5}• A_{+1}, A_{-4}• A_{+2} and T_{-3}• A_{+3} base pairs through trans Hoogsteen/Hoogsteen, trans Hoogsteen/Watson-Crick and trans Watson-Crick/Watson-Crick hydrogen bonds, respectively[1]. (a) CTD2* (yellow) complexed with ssDNA (blue) occupying an asymmetric unit (Molecule-A). Another CTD2*-ssDNA complex (gray) occupies the adjacent asymmetric unit (Molecule-B). Purple spheres represent Zn^{2+} ions. (b) An enlarged view of base pairing interaction between ss-DNA of Molecule-A (blue) and ssDNA of Molecule-B (gray).

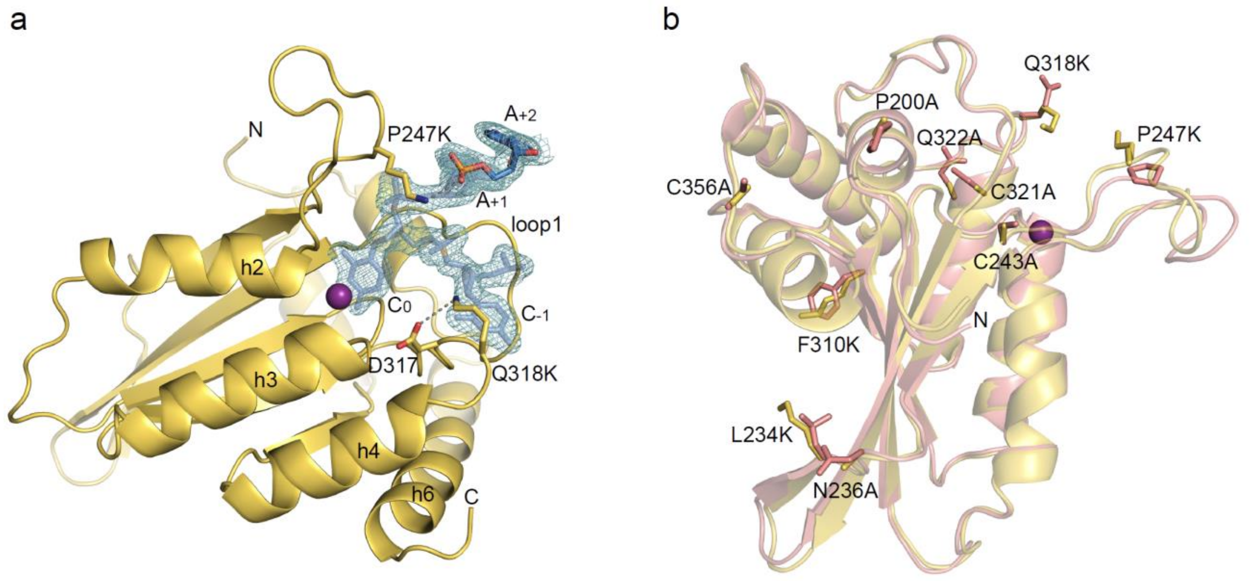

Supplementary Figure 4.

(a) P247K and Q318K substitutions provided additional supports to the CTD2*-ssDNA complex. Yellow cartoon shows the backbone structure of CTD2*. Side chains of P247K, D317, and Q318K shown in sticks, and N and O atoms are colored navy blue and red, respectively. Zn^{2+} ion is shown as a purple sphere. C_{-1}, C_{-0}, A_{+1} and A_{+2} nucleotides are displayed in blue stick, and P and O atoms of A_{+2} are colored orange and red, respectively. A 2Fo -Fc electron density map contoured at 1 σ is shown in cyan around the nucleotides. Gray dashed lines indicate a hydrogen bond between P247K and 5'-phosphate group of $A_{+2,}$ and an electrostatic interaction between Q318K and D317. (b) The ssDNA bound CTD2* structure (this study, yellow) is superimposed with a crystal structure of wild-type A3G-CTD (PDB ID#4ROV, pink). The ssDNA of the CTD2*-ssDNA complex is not shown. All amino acids substituted to generate CTD2, including P200A, L234K, N236A, C243A, P247K, F310K, Q318K, C321A, Q322A and C356A, are presented as sticks.

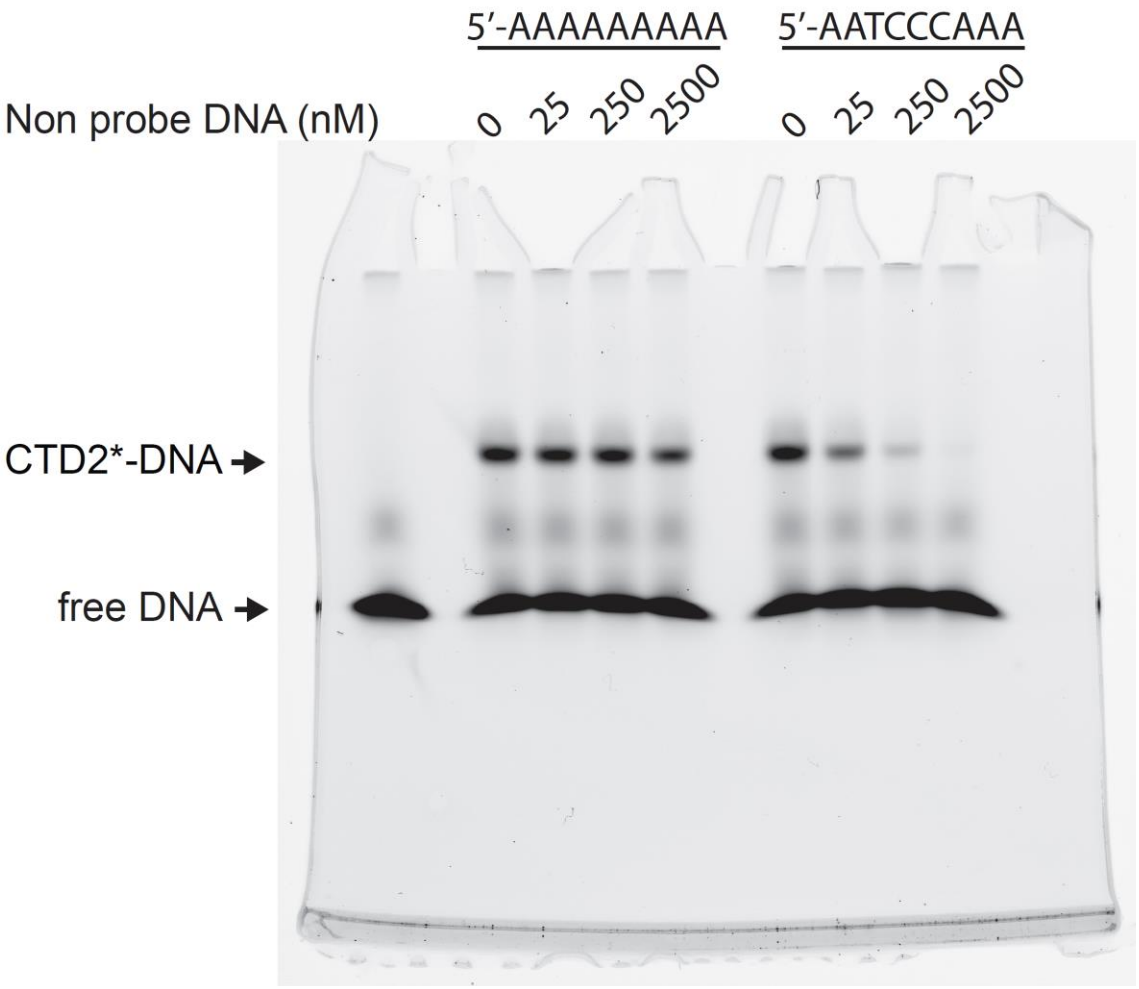

Supplementary Figure 5.

Full gel of EMSA for binding of CTD2* to the 9nt ssDNA (5′-AATCCCAAA-6-FAM) used to generate **Figure 1c**. CTD2*-DNA indicates position of the CTD2*-ssDNA complex, and free DNA indicates the position of protein-free ssDNA. Fluorescent-unprobed 9nt polyA (5′-AAAAAAAAA) or unprobed 9nt ssDNA (5′-AATCCCAAA) were added with incremental amounts in lanes 3 (25 nM), 4 (250 nM) and 5 (2500 nM) or 7 (25 nM), 8 (250 nM) and 9 (2500 nM), respectively.

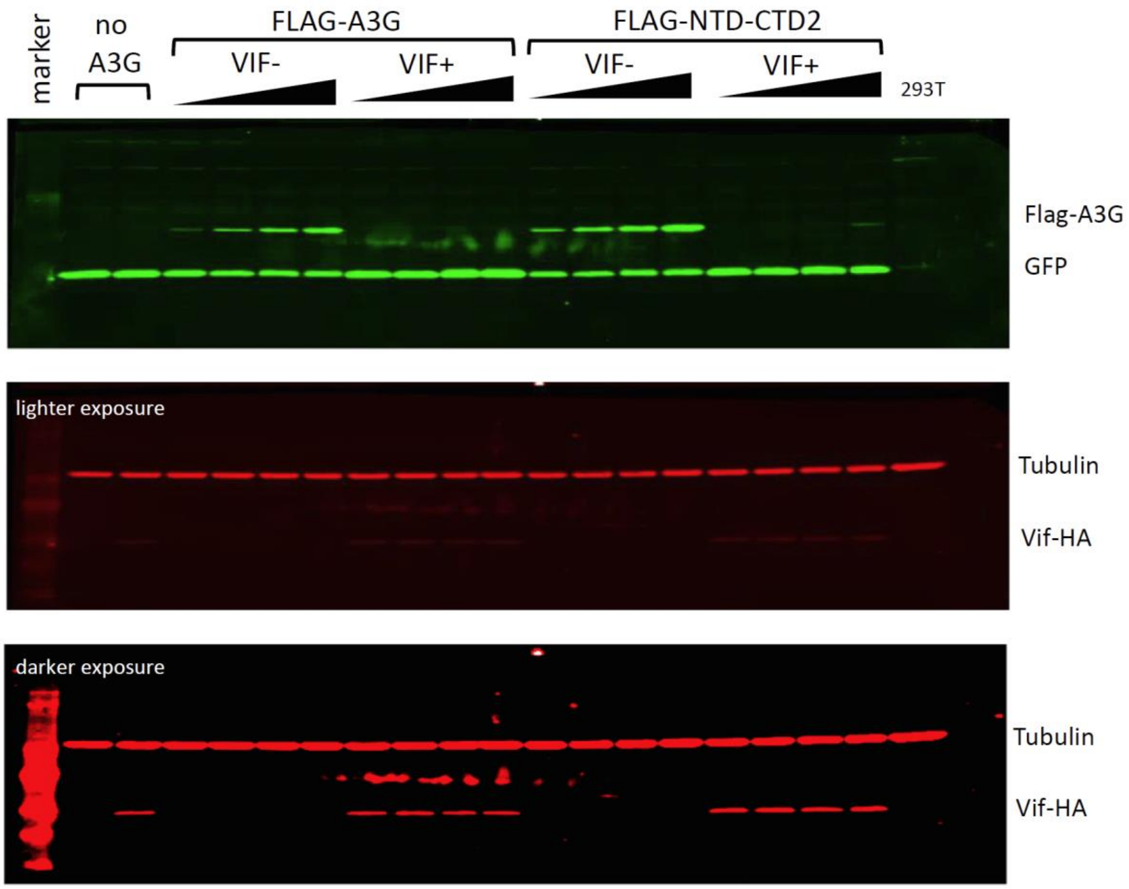

Supplementary Figure 6.

Original gel blots used to produce **Figure 2a**. Western blot showing 293T cells co-transfected with increasing amounts of wild-type FLAG-A3G or FLAG- NTD-CTD2 (21, 42, 84, 170 ng), HDV-EGFP and VSV-G in the presence or absence of Vif-HA.

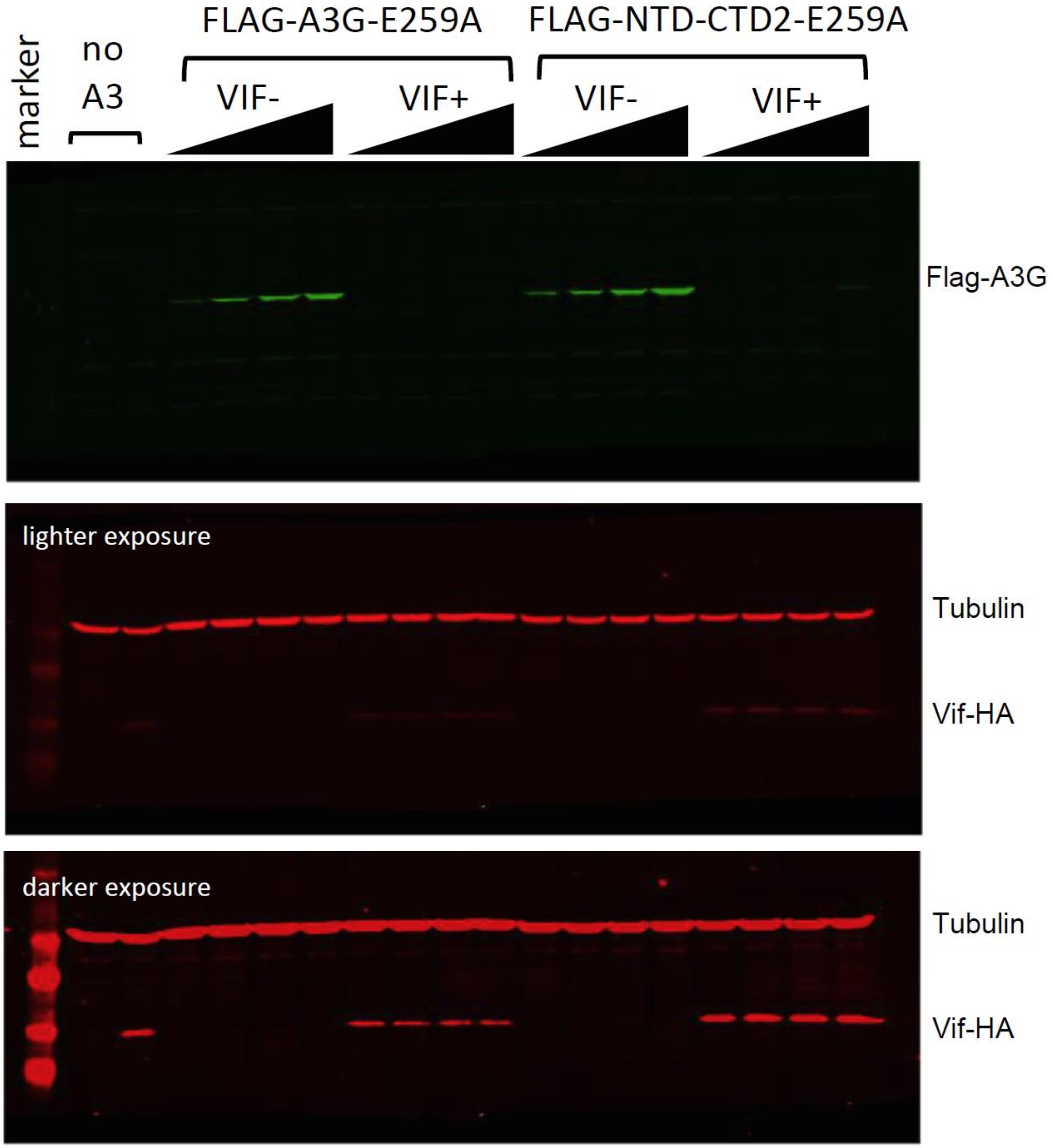

Supplementary Figure 7.

Original gel blots used to produce **Supplementary Figure 2a**. Western blot showing 293T cells co-transfected with increasing amounts of wild-type FLAG-A3G-E259A or FLAG-NTD-CTD2-E259A (21, 42, 84, 170 ng), HDV-EGFP and VSV-G in the presence or absence of Vif-HA.

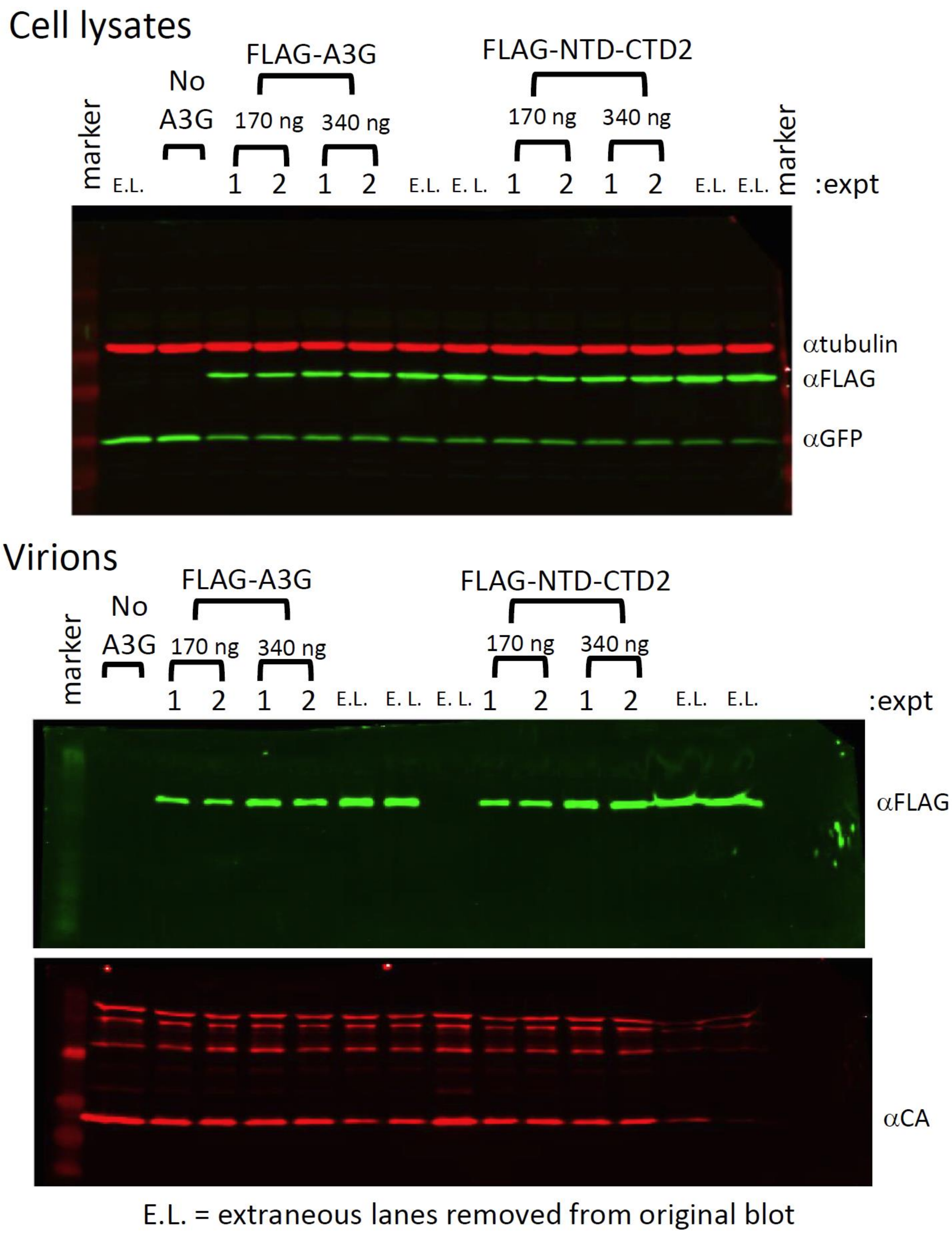

Supplementary Figure 8.

Original gel blots used to produce **Supplementary Figure 2d**. Western blot of 293T cell lysates and virions produced from 293T cells co-transfected with FLAG-A3G or FLAG-NTD-CTD2 (170 ng or 340 ng), HDV-EGFP and VSV-G in the absence of Vif-HA.

Supplementary Table 1

Substitution	Primer sequence
E259A-forward	5'-CCTGGAAGGTCGTCACGCAGCACTGTGCTTTCTGGATGTCA
E259A-reverse	5'-TGACATCCAGAAAGCACAGTGCTGCGTGACGACCTTCCAGG
Q318K-forward	5'- GCCCGCATCTATGATGATAAAGGAAGAGCTCAGGAGGGG
Q318K-reverse	5'- CCCCTCCTGAGCTCTTCCTTTATCATCATAGATGCGGGC

Supplementary References

1. Leontis, N.B., Stombaugh, J. & Westhof, E. The non-Watson-Crick base pairs and their associated isostericity matrices. *Nucleic Acids Res* **30**, 3497-531 (2002).
