# Supplementary material for: Crystal structure of the catalytic domain of HIV-1 restriction factor APOBEC3G in complex with ssDNA
Source: Nat Commun. 2018 Jun 25;9:2460. doi: 10.1038/s41467-018-04872-8 (PMC6018426; doi:10.1038/s41467-018-04872-8)
Supplement: Supplementary file 2 — Peer Review Report [file 41467_2018_4872_MOESM2_ESM.pdf]

Reviewers' comments:

Reviewer #1 (Remarks to the Author):

To date, many domain structures of the APOBEC3 (A3) cytidine deaminases have been determined by NMR or X-ray crystallography. Of these, only the complex structures of A3A, the A3B catalytic domain (CTD, C-terminal domain), and the A3F CTD that are bound to a piece of single-stranded DNA (ssDNA) substrate have been reported. However, the details of structural determinants for their substrate specificity have not been fully understood. In this study, Maiti et al. determined the first crystal structure of the A3G CTD complex bound to 9-nt ssDNA at 1.86å resolution.

First, the authors elaborated the A3G CTD variant, called “CTD2*”, that has a high binding affinity for ssDNA. In addition, the <i>in vitro</i> assay confirmed that the full-length A3G with ten residue substitutions equivalent to the CTD2* retained antiviral activity, similar to wild-type A3G. Using this variant, the authors obtained structure information of the A3G CTD-ssDNA complex. The data revealed that the CTD2* recognizes 5′-C₋₂-C₋₁-C ₀ nucleotides through hydrogen bonds formed on their Watson-Crick faces, and T₋₃ and A₊₁ via π-π interactions. Moreover, the A3G architecture consisted of the loops 1, 3, and 7, together with unique sugar puckers of the nucleotide substrate are likely to determine the specificity for ssDNA. These findings are unique and brings us new important insights to understand structure-based mechanisms of A3-mediated deamination reactions. Some modifications and corrections of the following points would significantly improve this manuscript.

Comments:
1) It is nicer for readers that the authors describe more clearly about major structural determinants for ssDNA specificity, though briefly stated about sugar pucker of nucleotides at pages 14-15. Can the authors comment whether there are spatial restraints at the C2′ of sugar for DNA or any steric hinderance for accommodation of RNA ribose 2′-hydroxyl groups in the substrate recognition site?
2) The authors’ finding that the A3G recognizes the Watson-Crick faces of 5′-C₋₂-C₋₁-C ₀ nucleotides is important. This supports some earlier observations that A3G efficiently deaminates substrates containing 5-methyl dC at C₋₁ or C₋₂, but inefficiently does ones containing 5-methyl dC at C₀ (for example, Rausch et al. JBC 284. 7047-). Additional discussion(s) about the specificity for modified nucleobases would improve the manuscript.
3) The authors do not sufficiently explain about the nucleobase preference at A₊₁ in text. The authors state that strongπ-π stacking between the purine at A₊₁ and H216 provides the preferential deamination sequence of the 5′-CCCA. It is unlikely that a base pair formation with A₋₅ of a neighboring asymmetric unit observed in the crystal would play an important role in the preference. Can the authors comment why A is preferred to G at the +1 position?

Minor comments:
4) Page 6. There is no information on P247K and Q318K in ref#23. The authors need to cite a correct reference regarding the P247K and Q318K. Otherwise, it should be explained why these two residues were chosen.
5) Figure 1. Mobility of the shifted bands is different between CTD2* and 2K3A* in EMSA. Is this because of five additional substitutions in CTD2*?
6) Figure 2 a and Supplementary Figure 2 a. Labeling “–Vif” and “+Vif” are necessary.
7) Figure 3 g. The authors need to indicate the interaction of P247K that increases ssDNA-binding affinity.
8) Page 32, line 7 from the bottom. Better to indicate “g” for centrifugation instead of “rpm”.
9) Page 37, lines 7-8 from the bottom. Insert the “p” prefix for each plasmid name.

Reviewer #2 (Remarks to the Author):

The manuscript by Maiti et al. describes a newly engineered C-terminal domain (CTD) of human APOBEC3G (A3G) with improved binding to single-stranded DNA. Binding is quantified by determination of Kd using MicroScale Thermophoresis (MST). The findings are novel and contribute considerably to the understanding of molecular interactions of APOBEC3G and related proteins with their nucleic acid substrate. It is interesting that a full length A3G protein containing a WT N-terminal domain (NTD) and the engineered CTD also inhibited HIV in cell-based assays in the absence of Vif. The high resolution crystal structure of the engineered CTD in complex with ssDNA reveals the details of how the CTD specifically recognizes ssDNA. The crystallographic data collection and refinement statistics are of excellent quality, and overall the manuscript is well written.
One question: have the authors checked whether the newly engineered CTD can also bind RNA with improved affinity?

Reviewer #3 (Remarks to the Author):

The manuscript by Maiti et al, “Crystal structure of catalytic domain of HIV-1 restriction factor A3G in complex with ssDNA” is potentially interesting work. Here they report a crystal structure of the C-terminal domain (CTD) of A3G containing 10 amino acid substitutions. Full length human A3G comes with two domains, namely NTD and CTD among them CTD is only catalytically active, but NTD essential for oligomerization, nucleic acid binding, and specific interaction with HIV-1 Vif. Although there are several high-resolution A3G-CTD structures, and some structures of APOBEC3 (A3A, A3B, rhesus A3G-NTD) proteins bound to ssDNA are available, A3G-CTD bound to ssDNA is absent. Not to mention, there is no full-length structure of A3 protein yet known.
In this study, the authors improved the ssDNA binding and catalytic activity by mutating five residues (CTD2) that previously had five solubility enhancing mutations (2K3A). The DNA binding capacity of these two mutants of A3G-CTD (without wild type!) was analyzed by different methods, the first complex crystal structure of A3G-CTD2*(“*”E259A, catalytically inactive) with 9mer ssDNA containing 5’-TCCCA motif was solved, and the HIV-1 Vif degradation of these variants tested. The experiments are technically well done and the manuscript in general, well written. However, in absence of both full-length or CTD apoenzyme structure (without ssDNA) and artificial mutations in the protein, the conclusion drawn to explain the binding mechanism is a bit of an exaggeration.
As stated, the A3G-CTD structures have been studied since 2008, except for these labs, others successfully made wild type structures (Holden et al 2008, Furukawa et al 2009, Lu et al 2015). A quick comparison of the 2K3A protein (pdb# 3IR2) surface with the wild type (pdb# 4ROV) shows difference between the loop structures, Arg-213 in 3IR2 also points towards the DNA binding groove, meaning the 2K3A has already an improved tendency to bind and act on DNA more than that of wild-type (Lu et al, Chen et al 2008). The extensive additional mutations that intended to render more basicity, and deamination activity, making the molecule more complex. Therefore, a structure of CTD2 without ssDNA complex is needed to compare and validate (molecular switching of) the DNA-protein interaction reported here.
Deamination activity using NMR (Fig 1, Suppl. Fig 1), EMSA, and thermophoresis experiments are missing the wild type enzyme (A3G-CTD). As reported before by the authors’ group, the 2K3A has 2.7 fold more catalytic function and 4 fold more solubility (Chen et al 2008), we don’t know the global effect of these 10 residues. Also, the quality of EMSA is not convincing, the mobility shift of protein-DNA complex catalytically inactive CTD2* and 2K3A* varies in length. Is it due to the difference in the molecular mass of two proteins (must be trivial)? Moreover, a negative control (unlabeled excess ssDNA that disrupt the complex) can be added to ensure specific interaction. As a note, full-length A3G interacts with ssDNA with no base specificity (Iwatani et al 2006).

Considering the difficulties in obtaining full-length protein for structure determination, I would definitely expect activity assay, EMSA, and if possible, MST with biologically relevant (full-length) purified protein (Iwatani et al 2006, and many others) and the 2K3, CTD2 variants could be made. This will strengthen the theme of the paper.
Figure 2, viral restriction assays are convincing (although viral packaging of A3G is not shown), relatively more expression of FLAG-NTD-CTD2, compared to the wild type might have resulted in more antiviral activity. This supports the previous comment that the full-length enzyme represents the real A3G which exist as multimeric complex in human cells (Chelico 2006, Bulliard et al., 2009; Huthoff et al., 2009; Salter 2009; Chaurasiya nature chem 2014) and it was shown that dimerization is important for A3G (McDougall 2011).

Minor comments
1. Abstract: The later part sentence start with, “A3G recognizes not only the 5’-CC motif but also..” can be modified to convey the specific message that the ssDNA containing CC motif is interacting with A3G. Considering the two-lysine mutations near N244, and since only one DNA ligand tested.
2. P4, end of first paragraph. I would take out the last sentence (or paraphrase) questioning the CC specificity and processivity of A3G. It was experimentally shown that loop 7, in particular D317 determine the CC to TC specificity of full-length-wild-type A3G (Rathore et al 2013)
3. P10, sentence, “This may indicate that the CTD2*-ssDNA complex is more stable at low temperature,….thermal motion” please add a citation here.
4. Discuss the importance of W211 with a forthcoming similar study (Ziegler et al) from a peer group (https://doi.org/10.1101/235481)

We would like to thank the reviewers for the time and attention dedicated to helping us improve our manuscript. We answered all comments point-by-point in the following sections. Reviewers comments are shown in italic font and highlighted in gray.

Reviewer #1 (Remarks to the Author):

Comments:

1) It is nicer for readers that the authors describe more clearly about major structural determinants for ssDNA specificity, though briefly stated about sugar pucker of nucleotides at pages 14-15. Can the authors comment whether there are spatial restraints at the C2′ of sugar for DNA or any steric hinderance for accommodation of RNA ribose 2′-hydroxyl groups in the substrate recognition site?

Thank you very much for this comment. We added following sentence in page 12-13.

"Although the CTD2*-ssDNA complex showed that C2′-endo sugar conformation of the target cytidine is required to fit in the catalytic pocket, the mechanism by which A3G discriminates RNA from deamination is not fully understood because the 2′ hydrogen of the target cytidine can be replaced with a hydroxyl group without significant steric hindrance in the CTD2*-ssDNA complex structure."

2) The authors' finding that the A3G recognizes the Watson-Crick faces of 5′-C_{-2}-C_{-1}-C_0 nucleotides is important. This supports some earlier observations that A3G efficiently deaminates substrates containing 5-methyl dC at C_{-1} or C_{-2}, but inefficiently does ones containing 5-methyl dC at C_0 (for example, Rausch et al. JBC 284. 7047-). Additional discussion(s) about the specificity for modified nucleobases would improve the manuscript.

Thank you very much for this comment. We added the following sentence in page 14.

"Previously, Rausch *et al*. showed that N3 and NH_2 of cytosine ring of C_{-1} and C_{-2} are important for deamination, whereas 5-methyl deoxy-cytidine at C_{-1} or C_{-2} position are tolerated by A3G, which is consistent with the CTD2*-ssDNA structure showing that Watson-Crick faces of C_{-1} and C_{-2} interact with CTD2*, while the C5 positions do not have a contact with the protein (**Fig. 3b**)."

3) The authors do not sufficiently explain about the nucleobase preference at A_{+1} in text. The authors state that strongπ-π stacking between the purine at A_{+1} and H216 provides the preferential deamination sequence of the 5′-CCCA. It is unlikely that a base pair formation with A_{-5} of a neighboring asymmetric

unit observed in the crystal would play an important role in the preference. Can the authors comment why A is preferred to G at the +1 position?

We agree with the reviewer. The CTD2*-ssDNA structure does not explain the preference of adenine over guanine at the +1 position because the Watson-Crick face of A_{+1} does not contact with CTD2*. Previously, Rausch *et al*. (*JBC*, pp7047-7058, 2009) showed that wild-type A3G efficiently deaminates a substrate containing a guanine at the +1 position. We also tested deamination of 5′-AATCCCGAA with CTD2, and found that CTD2 deaminates 5′-AATCCCGAA as efficient as 5′-AATCCCAAA since initial reaction rates were 10.56±0.24 and 10.92±0.48 reaction $minutes^{-1}$, respectively, at the same reaction condition, 200 µM ssDNA, 50 nM CTD2, and pH 6.5. Further studies are required to understand the preference of nucleotides at the +1 position.

Minor comments:
4) Page 6. There is no information on P247K and Q318K in ref#23. The authors need to cite a correct reference regarding the P247K and Q318K. Otherwise, it should be explained why these two residues were chosen.

P247K and Q318K substitutions were newly explored in this study. We changed a sentence in page 6 as following.

"We tested lysine substitutions of residues in loop1 and loop7 to increase the basic characteristics of the region near the active site, and P247K and Q318K were chosen because they enhanced catalytic activity. Those lysine substitutions were combined with the other three substitutions which we previously showed increased catalytic activity[23]."

5) Figure 1. Mobility of the shifted bands is different between CTD2 and 2K3A* in EMSA. Is this because of five additional substitutions in CTD2*?*

We think that additional two lysines on the surface of the CTD2* molecule may retard the mobility of CTD2* compared with the 2K3A.

6) Figure 2 a and Supplementary Figure 2 a. Labeling "–Vif" and "+Vif" are necessary.

We added "-Vif' and "+Vif" to **Fig. 2** and **Supplementary Fig. 2**.

7) Figure 3 g. The authors need to indicate the interaction of P247K that increases ssDNA-binding affinity.

We added P247K in **Fig. 3g**.

8) Page 32, line 7 from the bottom. Better to indicate "g" for centrifugation instead of "rpm".

We indicated as 48,384 *g*.

9) Page 37, lines 7-8 from the bottom. Insert the "p" prefix for each plasmid name.

We inserted "p" prefix for each plasmid.

Reviewer #2 (Remarks to the Author):

One question: have the authors checked whether the newly engineered CTD can also bind RNA with improved affinity?

As reviewer suggested, we tested the binding of ssRNA 5′-rArArUrCrCrCrArArA to CTD2* using MST, and K_d was determined to be 1.5 ± 0.5 mM (**Rebuttal Fig. 1**) that is 27 times greater than that of ssDNA substrate 5′-AATCCCAAA. We added following sentence describing this result in page 8.

"Furthermore, we tested CTD2* for binding a 9nt RNA 5′-rArArUrCrCrCrArArA, and K_d was determined to be 1.5 ± 0.5 mM (data not shown)."

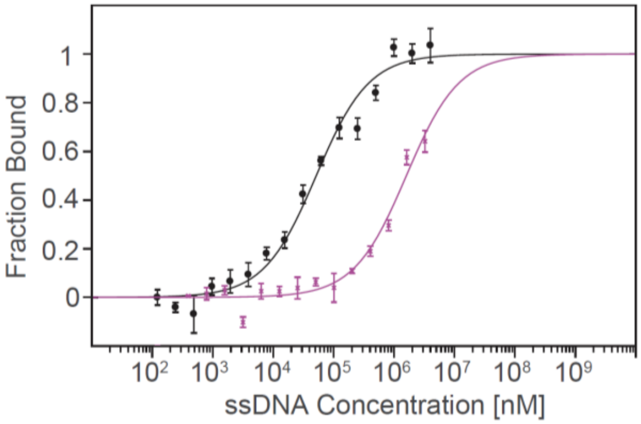

Rebuttal Figure 1. Each dot represents microscale thermophoresis (MST) measurement of a mixture containing fluorescent labeled CTD2* (50nM) and 9nt ssDNA 5′-AATCCCAAA (black) and 9nt ssRNA 5′-rArArUrCrCrCrArArA (magenta) at various concentrations including 0.12 µM, 0.24 µM, 0.48 µM, 0.97 µM, 1.95 µM, 3.90 µM, 7.81 µM, 15.62 µM, 31.25 µM, 62.5 µM, 125 µM, 0.250mM, 0.5mM, 1mM, 2mM and 4mM for the ssDNA, and 0.4 µM, 0.8 µM, 1.6 µM, 3.1 µM, 6.2 µM, 12.5 µM, 25 µM, 50 µM, 100 µM, 200 µM, 400 µM, 800 µM, 1.6 mM, and 3.2 mM for the ssRNA. Three independent MST experiments were performed, and bars of data points represent standard error of n=3 measurements.

Reviewer #3 (Remarks to the Author):

A quick comparison of the 2K3A protein (pdb# 3IR2) surface with the wild type (pdb# 4ROV) shows difference between the loop structures, Arg-213 in 3IR2 also points towards the DNA binding groove, meaning the 2K3A has already an improved tendency to bind and act on DNA more than that of wild-type (Lu et al, Chen et al 2008).

We respectfully disagree with this comment regarding that A3Gctd-2K3A has improved affinity for ssDNA compared with wild-type A3Gctd because the loop1 of CTD2 is completely wild-type sequence, and kinetic parameters of the deamination catalyzed by A3Gctd-2K3A are equivalent to those of wild-type A3Gctd (Harjes, *et al*., *JVI*, pp7008-7014, 2013).

The extensive additional mutations that intended to render more basicity, and deamination activity, making the molecule more complex. Therefore, a structure of CTD2 without ssDNA complex is needed to compare and validate (molecular switching of) the DNA-protein interaction reported here.

We respectfully disagree with this comment because the substitutions introduced to CTD2 did not alter the structure, and existing structures can validate our results. We added **Supplementary Figure 4b** showing all substitutions in superimposed structures of CTD2* (this study) and wild-type A3G-CTD (4ROV, Lu *et al*., *JBC*, 2015), then added following sentence in the revised manuscript (page 17).

"It is noteworthy that the substitutions introduced to CTD2 did not change the structure of A3G-CTD as shown in the superimposed structures of CTD2* (this study) and wild-type A3G-CTD (PDB ID: 4ROV) (**Supplementary Fig. 4b**). CTD2* and 4ROV structures are well superimposed as indicated by the

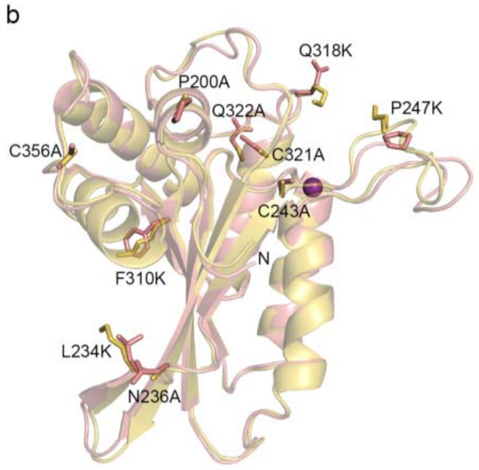

pairwise root mean square (rms) deviation, which is 0.9Å."

Supplementary Figure 4b. (b) The CTD2*-ssDNA complex structure (this study, yellow) is superimposed with a crystal structure of wild-type A3G-CTD (PDB ID#4ROV, pink). The ssDNA of the

CTD2*-ssDNA complex is not shown. All amino acids substituted in CTD2, including P200A, L234K, N236A, C243A, P247K, F310K, Q318K, C321A, Q322A and C356A, are presented as sticks.

Furthermore, the movement of loop1 upon ssDNA-binding can also be validated by comparisons of the CTD2*-ssDNA structure with apo-form wild-type A3Gctd structures including 4ROV (Lu *et al*., *JBC*, pp4010-4021, 2015) and 3IQS (Holden *et al*., *Nature*, pp121-124, 2008). **Rebuttal Fig. 2** shows the loop1 region of the superimposed structures of CTD2* (yellow), 4ROV (pink) and 3IQS (blue), and it shows rearrangement of loop1 residues including W211 and R213.

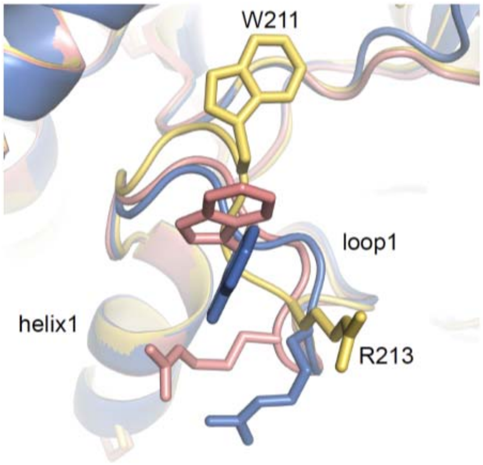

Rebuttal Figure 2. Superimposition of structures of wild-type A3Gctd (3IQS, blue), wild-type A3Gctd (4ROV, pink) and CTD2* (this study, yellow). Sidechains of W211 and R213 are shown in sticks.

Deamination activity using NMR (Fig 1, Suppl. Fig 1), EMSA, and thermophoresis experiments are missing the wild type enzyme (A3G-CTD). As reported before by the authors' group, the 2K3A has 2.7 fold more catalytic function and 4 fold more solubility (Chen et al 2008), we don't know the global effect of these 10 residues.

Since A3Gctd-2K3A is equivalent to wild-type A3Gctd in regard to enzymatic kinetics (Harjes, *et al*., *JVI*, pp7008-7014, 2013), we used A3G-CTD-2K3A in the ssDNA binding and deamination assays. The 2.7 fold increment in mutation frequency of A3G-CTD-2K3A is likely due to its improved solubility because the *E. coli*-based Rif^r mutation assay used in the reference (Chen *et al*., *Nature*, pp116-119, 2008) is sensitive to expression level and solubility of the enzyme.

Also, the quality of EMSA is not convincing, the mobility shift of protein-DNA complex catalytically inactive CTD2 and 2K3A* varies in length. Is it due to the difference in the molecular mass of two proteins (must be trivial)?*

We think that additional two lysines on the surface of the CTD2* molecule retard the mobility of CTD2*.

Moreover, a negative control (unlabeled excess ssDNA that disrupt the complex) can be added to ensure specific interaction.

We tested binding of CTD2* to the deamination products including 5′-AATCCdeoxy-UAAA, 5′-AATCdeoxy-Udeoxy-UAAA using MST, and determined their K_ds to be 150 ± 30 μM and 5.2 ± 0.8 mM, respectively. Note that 5′-AATCCdeoxy-UAAA is the substrate to produce 5′-AATCdeoxy-Udeoxy-UAAA, while 5′-AATCdeoxy-Udeoxy-UAAA will not be deaminated. Since the K_d value for 5′-AATCCCAAA is 55 μM (this study), the result clearly indicates that CTD2* specifically binds substrate ssDNAs compared with non-substrate ssDNA. We added **Supplementary Figure 1c** showing the MST assay results of 5′-AATCCdeoxy-UAAA and 5′-AATCdeoxy-Udeoxy-UAAA, and added following sentence in page 8.

"As expected from our previous studies[41,43], CTD2* appeared to have significantly less affinity to 5′-TCCdeoxy-UA (primary product) and 5′-TCdeoxy-Udeoxy-UA (secondary product) because K_d values for 9nt ssDNAs containing these product sequences were determined to be 150 ± 30 μM and 5.2 ± 0.8 mM, respectively (**Supplementary Fig. 1c**). "

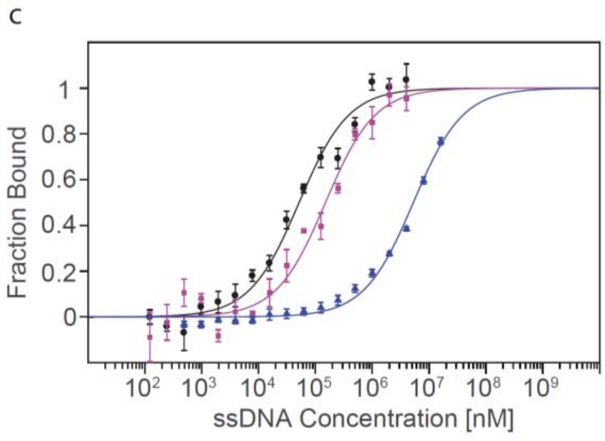

Supplementary Figure 1c.

(c) Each dot represents microscale thermophoresis (MST) measurement of a mixture containing fluorescent labeled CTD2* (50nM) and 9nt ssDNAs including 5′-AATCCCAAA (black), 5′-AATCCdeoxy-UAAA (magenta) and 5′-AATCdeoxy-Udeoxy-UAAA (blue) at various concentrations including 0.12 μM, 0.24 μM, 0.48 μM, 0.97 μM, 1.95 μM, 3.90 μM, 7.81 μM, 15.62 μM, 31.25 μM, 62.5 μM, 125 μM, 0.250mM, 0.5mM, 1mM, 2mM and 4mM. Additionally, we measured MST at 8mM and 16mM concentration for 5′-AATCdeoxy-Udeoxy-UAAA (blue). Three independent MST experiments were performed, and bars of data points represent standard error of n=3 measurements.

As a note, full-length A3G interacts with ssDNA with no base specificity (Iwatani et al 2006). Considering the difficulties in obtaining full-length protein for structure determination, I would definitely expect activity assay, EMSA, and if possible, MST with biologically relevant (full-length) purified protein

(Iwatani et al 2006, and many others) and the 2K3, CTD2 variants could be made. This will strengthen the theme of the paper.

We understand the importance of studying wild-type protein, but wild-type full-length A3G is inhomogeneous in regard to oligomeric states even at μM concentration, which prevents quantitative analysis of the biophysical assays used in our study.

Figure 2, viral restriction assays are convincing (although viral packaging of A3G is not shown), relatively more expression of FLAG-NTD-CTD2, compared to the wild type might have resulted in more antiviral activity. This supports the previous comment that the full-length enzyme represents the real A3G which exist as multimeric complex in human cells (Chelico 2006, Bulliard et al., 2009; Huthoff et al., 2009; Salter 2009; Chaurasiya nature chem 2014) and it was shown that dimerization is important for A3G (McDougall 2011).

We agree with the reviewer that the slightly higher expression of the Flag-NTD-CTD2 likely resulted in higher antiviral activity. We understand that oligomerization of A3G affects its functions. Most recently, Morse *et al.* studied oligomerization of A3G by using single-molecule methods, and they showed that monomeric A3G is catalytically active, whereas dimerization and higher oligomerization reduce A3G deamination efficiency (Morse *et al.*, *Nat. Commun*. 597, 2017). Therefore, the monomeric structure of CTD2* complexed with a ssDNA is important to understand the catalytic mechanism of wild-type A3G.

Minor comments
1. Abstract: The later part sentence start with, "A3G recognizes not only the 5'-CC motif but also.." can be modified to convey the specific message that the ssDNA containing CC motif is interacting with A3G. Considering the two-lysine mutations near N244, and since only one DNA ligand tested.

We changed the sentence to be read as

"This structure reveals atomic-level interactions by which APOBEC3G recognizes a functionally-relevant 5′-TCCCA sequence."

2. P4, end of first paragraph. I would take out the last sentence (or paraphrase) questioning the CC specificity and processivity of A3G. It was experimentally shown that loop 7, in particular D317 determine the CC to TC specificity of full-length-wild-type A3G (Rathore et al 2013)

We deleted the section "nonetheless, the underlying mechanism for this processivity remains elusive.", so the new sentence reads as following.

"A3G's deamination mechanism may be more complicated than that of other A3 proteins because several groups have reported that A3G deaminates 5′-CC hotspots processively from the 3′-end to the 5′-end of ssDNA[22]".

3. P10, sentence, "This may indicate that the CTD2-ssDNA complex is more stable at low temperature,….thermal motion" please add a citation here.*

This sentence is a speculation, and it is not supported by experimental data, therefore we deleted the sentence in the revised version (page 9).

4. Discuss the importance of W211 with a forthcoming similar study (Ziegler et al) from a peer group (https://doi.org/10.1101/235481)

This is a pre-submission manuscript deposited to BioRxiv. Since it has not been published, we feel that it is premature to discuss details of its contents. Furthermore, the structure described in this manuscript does not show specific interactions recognizing the target sequence of the deamination catalyzed by A3G.

Reviewers' comments:

Reviewer #1 (Remarks to the Author):

I think the authors appropriately revised the manuscript. In addition, I did not see any problem on the structure validation report.

Reviewer #3 (Remarks to the Author):

The authors submitted their revised text and I appreciate their efforts.
I still have some the following concerns:
It is known that A3 proteins possess a canonical fold and so a superimposition of A3G with other A3s such as A3F-CTD (it is RMSD 0.5 Å! between 4rov and 4iou) or A3C will be nicely aligning. So It is not surprising that CTD2* and wild-type A3G-CTD (RMSD 0.9 Å). Unfortunately, this will not be supporting to conclude that the mutated A3G (or A3F) align well with the overall structure and so they will interact and catalyze their substrate DNA in a similar manner (A3F does not prefer CCC). If not wild-type A3G, a structure that do not have DNA is still appreciated to have here.
It is very important to use the wild-type enzyme wherever possible (for deamination/EMSA/thermophoresis), and the mutants CTD2/2K3A may be compared together. Please consider including wild-type enzyme. These methods are fairly established by many groups.
New supplementary Fig 1c showing MST does not help to answer the problem in EMSA, they are two different methods. Either take out EMSA figure and show one method clearly with proper controls or add the negative control to the EMSA gel as suggested before. Using wild-type A3G in parallel would be always a great idea.
If you are not using the full-length enzyme for crucial methods (activity, EMSA, MST assays), how can you conclude that it is physiologically relevant? Getting inhomogeneous A3G is a known issue, and can be tackled by size exclusion chromatography or please refer Polevoda et al 2015, Iwatanti et al 2006. In case of failure, at least A3G-CTD have to be used as suggested above.
Please include the A3G virus encapsidation in parallel to infectivity assay to avoid any uncertainties.

We would like to thank the reviewers for insightful comments, which significantly improved our manuscript. We answered the remaining concerns of the reviewer #3 point-by-point in the following sections. Reviewers comments are shown in italic font and highlighted in gray.

Reviewer #3 (Remarks to the Author):

It is known that A3 proteins possess a canonical fold and so a superimposition of A3G with other A3s such as A3F-CTD (it is RMSD 0.5 Å! between 4rov and 4iou) or A3C will be nicely aligning. So It is not surprising that CTD2 and wild-type A3G-CTD (RMSD 0.9 Å). Unfortunately, this will not be supporting to conclude that the mutated A3G (or A3F) align well with the overall structure and so they will interact and catalyze their substrate DNA in a similar manner (A3F does not prefer CCC). If not wild-type A3G, a structure that do not have DNA is still appreciated to have here.*

We agree that the ssDNA-free structure of CTD2 would provide valuable information. Although we have not obtained the ssDNA-free structure of CTD2, we can answer the concerns raised by the reviewer.

Supplementary Figure 4b shows well aligned overall structures of CTD2* (this study) and a wild-type A3G-CTD (4ROV). This figure shows that the 10 mutations introduced in CTD2 did not change protein structure because not only Cα but also Cβ atoms of the mutated residues are well superimposed between CTD2* (yellow sticks) and 4ROV (pink sticks). The only exception is P247K which is located in an intrinsically disordered loop3. Indeed, loop3 shows different conformations in all known A3G-CTD structures.

We carefully evaluated whether CTD2 catalyzes substrate ssDNAs in a same manner as the wild-type A3G. We have shown that CTD2 has specific affinity to substrate ssDNAs by presenting K_d values of CTD2* for 5′-AATCCCAAA (substrate), 5′-AATCCdeoxy-UAAA (the first product) and 5′-AATCdeoxy-Udeoxy-UAAA (the final product), which are 55 µM, 150 µM and 5.2 mM, respectively (**Supplementary Figure 1c)**. In addition, we revised **Supplementary Figure 1a** to show entire course of deamination catalyzed by CTD2. CTD2 first deaminates the 3′ C in a 5′-CCC motif to produce 5′-CCdeoxyU, then the middle C in 5′-CCdeoxyU is deaminated to produce 5′-CdeoxyUdeoxyU, and the 5′ C is not acted upon by CTD2. This deamination manner has been well studied with wild-type A3G as well as A3G-CTD (Rausch *et al. JBC*, pp7047-7058, 2009) (Harjes, *et al.*, *JVI*, pp7008-7014, 2013) (Chiba *et al.*, Chem Commun, pp 12115-12117, 2012). Furthermore, this deamination assay affirms that CTD2 does not deaminate cytosine in the 5′-TC sequence because 5′-AATdeoxyUCCAAA or 5'-AATdeoxyUdeoxyUdeoxyUAAA was not produced. Therefore, ssDNA binding data and the deamination assays unambiguously indicate that CTD2 binds and catalyzes ssDNA substrates in the same manner as wild-type A3G does.

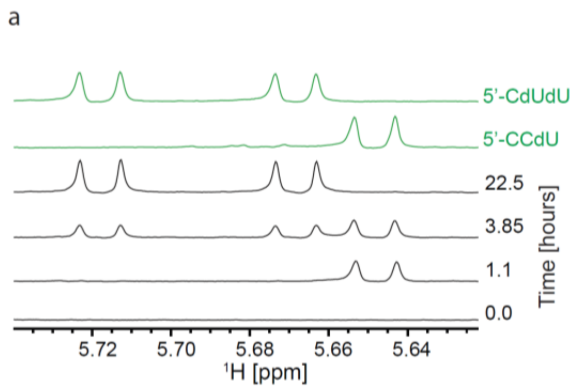

Revised Supplementary Figure 1a.
An enlarged region of a ^{1}H spectrum of the substrate 5′-AATCCCAAA is compared at several time points during deamination reactions to illustrate the increase in the H5 proton signal of uracil due to the deamination of the 3′ C producing 5′-AATCCdeoxy-UAAA (a doublet signal centered at 5.65ppm at pH 6.5) followed by the deamination of the central C producing 5′-AATCdeoxy-Udeoxy-UAAA (a doublet signal centered at 5.72ppm at pH 6.5). Spectra of 5′-AATCCdeoxy-UAAA and 5′-AATCdeoxy-Udeoxy-UAAA are provided as references (green spectra labeled 5′-CCdU and 5′-CdUdU, respectively). Due to the change in chemical environment, after formation of the 2nd deoxy-U, the position of the 1st deoxy-U H5 proton signal shifts from 5.65 ppm to 5.67ppm.

New supplementary Fig 1c showing MST does not help to answer the problem in EMSA, they are two different methods. Either take out EMSA figure and show one method clearly with proper controls or add the negative control to the EMSA gel as suggested before.

As the reviewer #3 suggested, we conducted EMSA with two controls including fluorescent-unprobed non-specific polyA ssDNA (5′-AAAAAAAAA) and fluorescent-unprobed specific ssDNA (5′-AATCCCAAA) (**revised Figure 1c**). The CTD2*-ssDNA band intensity was not affected by 5′-AAAAAAAAA, while the band intensity reduced in proportion to the amount of the unprobed 5′-AATCCCAAA. Therefore, new EMSA experiment shows the specific affinity of CTD2* for the substrate 5′-AATCCCAAA. Text was revised to refer the **revised Figure 1c** in pages 7 and 8.

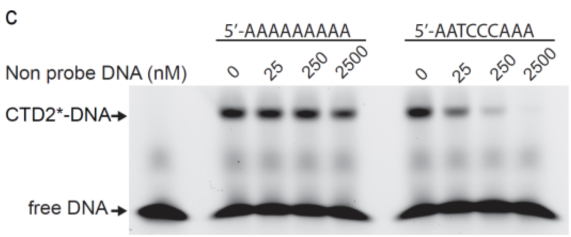

Revised Figure 1c.
EMSA for binding of CTD2* to the 9nt ssDNA (5′-AATCCCAAA-6-FAM). CTD2*-DNA indicates position of the CTD2*-ssDNA complex, and free DNA indicates the position of protein-free ssDNA. Fluorescent-unprobed 9nt polyA (5′-AAAAAAAAA) or fluorescent-unprobed 9nt ssDNA (5′-AATCCCAAA) were added with incremental amounts in lanes 3 (25 nM), 4 (250 nM) and 5 (2500 nM) or 7 (25 nM), 8 (250 nM) and 9 (2500 nM), respectively.

If you are not using the full-length enzyme for crucial methods (activity, EMSA, MST assays), how can you conclude that it is physiologically relevant? Getting inhomogeneous A3G is a known issue, and can be tackled by size exclusion chromatography or please refer Polevoda et al 2015, Iwatanti et al 2006. In case of failure, at least A3G-CTD have to be used as suggested above.

As the reviewer #3 suggested, we used wild-type A3G-CTD in the deamination assays (**revised Figure 1b** and **Supplementary Figure 1b**). Initial speed of deamination of wild-type A3G-CTD was 0.3 ± 0.1 reactions min^{-1} at pH 7.5, which is 20 times slower than that of CTD2.

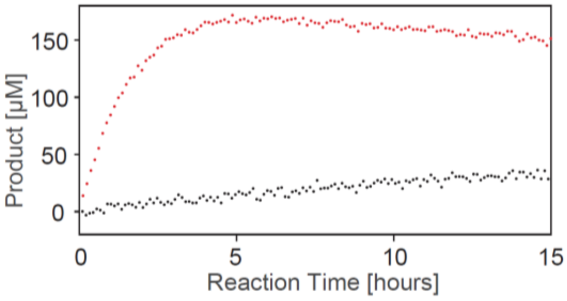

Revised Figure 1b.
Real-time NMR deamination assay. The product (5′-AATCCdeoxy-UAAA) concentration as a function of reaction time is plotted for CTD2 (red dots) and wild-type A3G-CTD (black dots) deamination at pH 7.5 with 200 nM protein and 200 µM 5′-AATCCCAAA substrate. For CTD2, the first reaction reached completion within 5 hours, and the second deamination (5′-AATCCdeoxy-UAAA to 5′-AATCdeoxy-Udeoxy-UAAA) started, thus decreasing the concentration of the initial product.

In addition, we performed EMSA and MST experiments using catalytically inactive wild-type A3G-CTD (A3G-CTD*) as the reviewer #3 suggested. Both experiments showed that A3G-CTD* has very weak affinity for the substrate 5′-AATCCCAAA. We added new **Supplementary Figure 1d** that shows a faint shifted-band observed with 160 µM of A3G-CTD*, consistent with previously reported observations by many laboratories. Text was revised to refer the **Supplementary Figure 1d** in page 8.

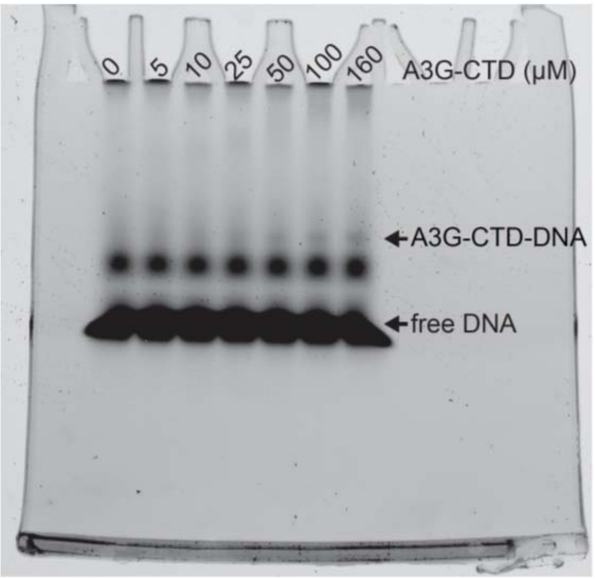

Supplementary Figure 1d.
EMSA for binding of A3G-CTD* to the 9nt ssDNA (5′-AATCCCAAA-6-FAM). A3Gctd-DNA indicates position of the A3G-CTD*-ssDNA complex, and free DNA indicates the position of protein-free ssDNA. 10 nM 5′-AATCCCAAA-6-FAM was incubated with with incremental amounts of A3G-CTD*, including 0, 5, 10, 10, 25, 50, 100 and 160 μM in lanes 1, 2, 3, 4, 5, 6 and 7, respectively.

Please include the A3G virus encapsidation in parallel to infectivity assay to avoid any uncertainties.

Virus encapsidation is now included in infectivity assays as **Supplementary Figure 2d and 2e** as the reviewer #3 suggested. Wild-type A3G and wild-type A3Gntd-CTD2 showed similar virus encapsidation. Following sentence was added in page 9 of the text. "Furthermore, no significant differences in viral infectivities were observed for virions encapsidating similar levels of FLAG-A3G or FLAG-NTD-CTD2 (t-test, $p > 0.3$) (**Supplementary Fig. 2d and 2e**)."

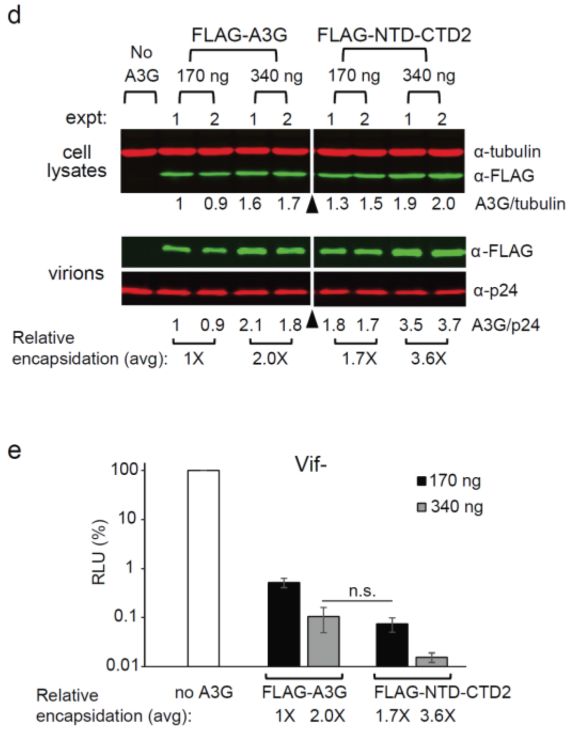

Supplementary Figure 2d and 2e.
(d) Representative western blot of 293T cell lysates and virions produced from 293T cells co-transfected with FLAG-A3G or FLAG-NTD-CTD2 (170 ng or 340 ng), HDV-EGFP and VSV-G in the absence of Vif-HA. FLAG-A3G or FLAG-NTD-CTD2 expression was normalized to tubulin levels in cell lysates and capsid p24 levels in virions. Fold change in expression levels is shown below each respective lane normalized to FLAG-A3G (170 ng, Expt 1). (e) Single-cycle infectivity of HDV-EGFP virus prepared in the presence of increasing amounts of FLAG-A3G or FLAG-NTD-CTD2 assayed in TZM-bl target cells. Data reflects the average relative light units (RLU) normalized to the no A3G control. Error bars represent the standard deviation for four independent infections from two viral preps. n.s., not significant (t-test, $p >$ 0.3). Black triangle, lanes derived from the same blot with

Most recently Ziegler *et al*. published a manuscript describing the structure of A3G-CTD bound to an adenine nucleotide (Ziegler *et al*., PLOSone, pp1-18, 2018). As reviewer #3 suggested in previous comment, we added following sentences describing structural differences between the CTD2*-ssDNA complex and the A3G-CTD-adenine complex in page 17.

"Most recently, Ziegler *et al*. published a structure of A3G-CTD bound to an adenine nucleotide[49]. In the A3G-CTD-adenine complex strcucture, the adenine nucleotide binds in the space that is similar to the T_{-1} position found in the A3A-ssDNA complexes[29,39]. Since C_{-1} in the CTD2*-ssDNA complex does not occupy the T_{-1} position (**Fig. 4c**), the protein-DNA interactions found in the A3G-CTD-adenine complex[49] are different from the enzyme-substrate interaction revealed in our study. Ziegler *et al*. suggested that the A3G-CTD-adenine structure shows a non-specific interaction by which A3G-CTD scans ssDNA sequence[49]. Iinterestingly, the W211 rearranged its position to interact with the bound adenine[49], which may imply that W211 is key in the interaction with non-specific DNA as well as the deamination target sequence."
